# Inflammation in Myocardial Ischemia/Reperfusion Injury: Underlying Mechanisms and Therapeutic Potential

**DOI:** 10.3390/antiox12111944

**Published:** 2023-10-31

**Authors:** Jamie Francisco, Dominic P. Del Re

**Affiliations:** Department of Cell Biology and Molecular Medicine, Cardiovascular Research Institute, Rutgers New Jersey Medical School, Newark, NJ 07103, USA

**Keywords:** ischemia/reperfusion, myocardial infarction, inflammation, macrophage, neutrophil

## Abstract

Acute myocardial infarction (MI) occurs when blood flow to the myocardium is restricted, leading to cardiac damage and massive loss of viable cardiomyocytes. Timely restoration of coronary flow is considered the gold standard treatment for MI patients and limits infarct size; however, this intervention, known as reperfusion, initiates a complex pathological process that somewhat paradoxically also contributes to cardiac injury. Despite being a sterile environment, ischemia/reperfusion (I/R) injury triggers inflammation, which contributes to infarct expansion and subsequent cardiac remodeling and wound healing. The immune response is comprised of subsets of both myeloid and lymphoid-derived cells that act in concert to modulate the pathogenesis and resolution of I/R injury. Multiple mechanisms, including altered metabolic status, regulate immune cell activation and function in the setting of acute MI, yet our understanding remains incomplete. While numerous studies demonstrated cardiac benefit following strategies that target inflammation in preclinical models, therapeutic attempts to mitigate I/R injury in patients were less successful. Therefore, further investigation leveraging emerging technologies is needed to better characterize this intricate inflammatory response and elucidate its influence on cardiac injury and the progression to heart failure.

## 1. Introduction: Myocardial Ischemia/Reperfusion Injury

Heart disease, often a result of myocardial infarction (MI), remains the leading cause of mortality worldwide [1,2,3,4]. Acute MI results from obstruction of the coronary arteries that supply the myocardium with blood; therefore, timely reperfusion (typically via percutaneous coronary intervention; PCI) is critical to preserving myocardial integrity and is considered the current gold standard treatment for MI patients [1,5]. Paradoxically, therapeutic reperfusion causes additional injury through several mechanisms, including rapid changes in pH, Ca^2+^ overload, and hyperoxia, leading to altered metabolism, the reversal of surface ion pumps, mitochondrial dysfunction/ROS production, and opening of the mitochondrial permeability transition pore (mPTP) [3,5,6,7,8,9,10]. This presents clinically as myocardial stunning, arrhythmias, and lethal reperfusion injury, in which salvageable cardiomyocytes in the area at risk undergo necrosis and/or additional forms of regulated cell death [3,7,8,9,11]. Thus, reperfusion directly contributes to infarct expansion and is now believed to account for up to half of the total infarct size [1,3,7,8,9,12]. In addition to MI, heart transplantation, which is the only therapeutic option for end-stage heart failure, is another setting for cardiac ischemia/reperfusion (I/R) injury, and I/R injury limits transplant effectiveness [3,5]. Despite its recognition as a significant contributor to myocardial damage following ischemia, reperfusion injury remains without approved therapeutic intervention and novel approaches to ameliorate this disease modality are needed [1,3,7,8,9,12]. Although a sterile environment, I/R initiates a complex inflammatory response that plays an important role in modulating the extent of cardiac injury and repair. The objectives of this review are to provide a detailed analysis of inflammatory cell functions as they relate to our current understanding of the pathology of acute MI, and to highlight studies that target inflammation therapeutically for cardiac I/R injury in preclinical models and patients.

## 2. Initiation of Inflammation in Cardiac I/R Injury

Both the initial injury due to ischemia and collateral damage imposed by reperfusion result in a massive loss of cardiomyocytes within the heart, and thus the release of damage-associated molecular patterns (DAMPs) from the infarcted myocardium [5,7,10,12,13,14,15]. These include nuclear (e.g., HMGB1), cytosolic (e.g., RNA), extracellular matrix (e.g., fibronectin), mitochondrial (e.g., mtDNA), and contractile (cardiac myosin) components of the myocardium [1,5,6,7,8,9,10,12,13,14,15,16,17,18]. Mechanistically, many of these DAMPs serve as ligands for pattern recognition receptors (PPRs), including Toll-like receptors (TLRs), NOD-like receptors (NLRs), receptors for advanced glycation end product (RAGE), and complement receptors, which are broadly expressed in the heart and known to facilitate I/R injury through signaling in multiple cell types [5,16,19,20,21,22,23,24,25,26].

**Figure 1:**
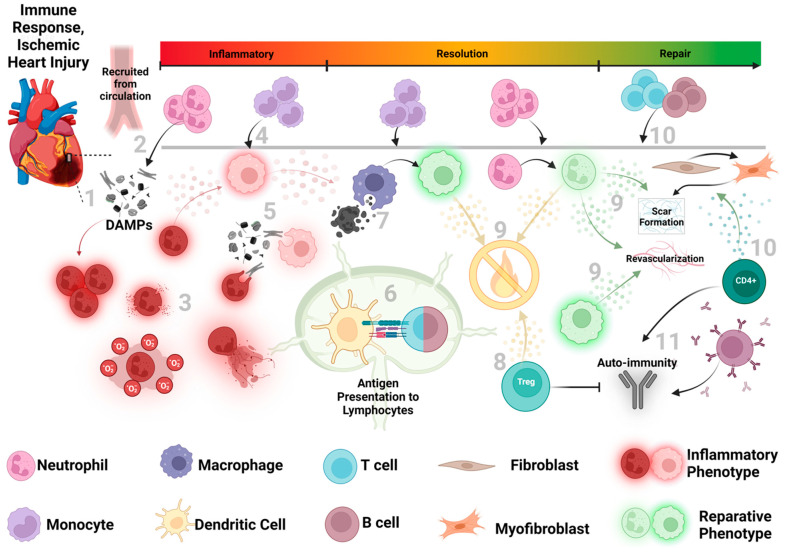
Overview of the immune response to ischemic heart injury (1) After acute MI, damage-associated molecular patterns (DAMPs) are released from the infarcted myocardium and initiate the immune response. (2) Neutrophils are rapidly recruited from the circulation, and upon arrival to the injured myocardium, polarize to an inflammatory phenotype, initiating the inflammatory phase (shown in red). (3) Inflammatory neutrophils release ROS, NETs, inflammatory cytokines/chemoattractants via degranulation, and begin clearing debris. (4) Recruited monocytes arrive at the site of injury and (5) differentiate into inflammatory macrophages, aiding in the clearance of debris and additional leukocyte recruitment via the production of inflammatory cytokines and chemokines. (6) Dendritic cells begin to activate the adaptive immune response by presenting antigens to lymphocytes in mediastinal lymph nodes. (7) The transition to resolution (shown in yellow), is promoted by macrophage efferocytosis of dying neutrophils, which initiates a phenotypic switch toward repair and resolution. (8) Treg release of anti-inflammatory signaling also facilitates resolution. (9) Further recruitment of neutrophils and macrophages now assume a reparative phenotype (shown in green), releasing anti-inflammatory/reparative mediators to further resolve inflammation, as well as pro-angiogenic and fibrotic factors to stimulate repair processes and scar stabilization. (10) Lymphoid cells infiltrate the injured myocardium and can contribute to fibroblast activation and scar formation. (11) The presentation of autoantigens to lymphocytes elicits an autoimmune response, delaying repair in the chronic phase of stable scar formation. Dysregulation of any phase of the immune response results in defective scar formation and impaired cardiac function. Importantly, this sequence of events is accelerated in reperfused MI (I/R) compared to non-reperfused MI.

The binding of DAMPs to PRRs increases the expression of proinflammatory cytokines and chemokines that recruit innate immune cells from the bone marrow and spleen to the site of injury in a process referred to as sterile inflammation, or inflammation in the absence of pathogens [1,5,6,7,8,9,12,13,14,15,16,17,18]. The engagement of TLR2 and 4, as well as RAGE, promote the activation of NF-κB to upregulate proinflammatory gene expression and prime the NLRP3 inflammasome, while signaling via TLR3 and 9 activate cGAS-STING and NF-κB to propagate a type 1 interferon response [5,10,27,28]. Interestingly, cardiac resident cells uniquely contribute to I/R- induced inflammation. Following injury, cardiomyocytes, cardiac fibroblasts, and resident macrophages release inflammatory and chemoattractant molecules, such as IL-1β, TNFα, IL-6, and CCL2, to generate a chemotactic gradient and recruit inflammatory myeloid cells to the infarct region [3,5,10,29]. IL-1β also mediates paracrine effects by upregulating the expression of adhesion molecules needed for immune cell extravasation and collagenases in the fibroblast to delay repair, while decreasing cardiomyocyte contractility through L-type channel uncoupling and increased ROS, worsening cardiac outcomes [19,30]. The endothelium weakens its cell junctions and upregulates selectins and cell adhesion molecules to facilitate leukocyte extravasation into the injured tissue [13]. Cardiac fibroblasts secrete granulocyte-macrophage stimulating factor (GM-CSF) and chemoattractants including CCL2, CCL7, and CXCL1, which stimulates the recruitment of myeloid cells and initiates their proliferation and differentiation in the bone marrow during emergency hematopoiesis [13,20,31,32,33,34]. Taken together, the release of DAMPs during I/R trigger PRR signaling that initiates the inflammatory response in multiple cell types within the myocardium, generating a chemoattractant gradient to promote leukocyte recruitment to the heart [4,32,33] (Figure 1).

## 3. Neutrophils

### 3.1. Neutrophil Priming

The first myeloid cells recruited to the heart after I/R are neutrophils, which are defined as short-lived myeloid-derived granulocytes and represent over half of all human leukocytes in circulation [35,36,37,38,39]. Under homeostatic conditions, neutrophils exist in a quiescent state and follow circadian-mediated release from the bone marrow into circulation via the reciprocal regulation of CXCR2/CXCR4 [36,37,38,39]. In response to I/R, neutrophils mobilize within minutes to hours along the chemoattractant gradient of cytokines and cellular debris to the injured heart [16,31,39,40,41,42,43,44]. This rapid status change in response to environmental cues is essential for neutrophil function as an immune first responder [36,37]. This “ready” state, known as priming, was shown to enhance neutrophil effector functions, including generation of ROS, release of neutrophil extracellular traps (NETs), degranulation, chemotaxis and adhesion, phagocytosis, synthesis of inflammatory mediators, and survival [36,37]. Neutrophil priming is initiated by exposure to DAMPs induced by I/R (e.g., TNFα, GM-CSF) [36,37], and is marked by the shedding of CD62L and subsequent increased surface expression of CD11b, CD18, CD66, and β2 integrins. Priming also mobilizes secretory granules as well as the NOX2 complex to the plasma membrane to facilitate rapid ROS generation upon activation [45]. Enhanced NETosis and phagocytotic capacitance were also observed in primed neutrophils, but the underlying mechanisms are not fully understood [36,37]. Although previously thought to be terminally differentiated and transcriptionally inactive, it is now appreciated that neutrophils are highly plastic and modulate gene expression governing key effector functions via engagement and activation of transcription factors NF-κB, C/EBP, CREB, HIF-1α, and MYC [35,36,37,46]. Primed neutrophils increase the synthesis and subsequent release of inflammatory mediators including IL-1α, IL-1β, IL-6, TNFα, among others. Interestingly, a slow regression of superoxide burst and CD11b upregulation in primed neutrophils over time was observed ex vivo, suggesting the ability to “de-prime”, or revert to a quiescent state, perhaps to minimize nonspecific tissue damage [47]. However, whether this aspect of neutrophil function can be leveraged therapeutically to mitigate cardiac damage caused by I/R remains unexplored.

### 3.2. Neutrophil Activation

The ability of neutrophils to transition to varied states of activation in vivo, and their resulting heterogeneity in response to MI, has only recently begun to be defined and appreciated. Elegant studies employing single-cell RNA sequencing observed markers of neutrophil priming and activation that are time-dependent in response to MI in mice, including altered CD62L, activation of C/EBP and HIF-1α, and gene expression signatures indicative of proinflammatory status [46,48]. Additional evidence was generated in models of ischemic heart failure, including time-dependent alterations in neutrophil gene expression, enhanced release of ROS, NETs, cytokine secretion, and enhanced neutrophil lifespan in infarcted tissue [35,36,37,39,41,46,49,50,51]. Genetic manipulation of neutrophils can also modulate their priming and activation status. In one such study, neutrophils were engineered to express a common gain-of-function JAK2(V617F) mutation. This resulted in elevated basal priming, as indicated by the constitutive phosphorylation of p47phox (subunit of NOX2) in these cells [36,37,51]. In response to acute MI, mice expressing mutant JAK2(V617F) had augmented inflammation and enlarged infarcts compared to WT controls, suggesting that enhanced neutrophil priming and activation exacerbates cardiac injury [49]. Reduced effector functions demonstrated by unprimed neutrophils coupled with the potential to de-prime these cells highlight the potential of this response for therapeutic intervention and warrants further investigation in I/R injury [36,37].

### 3.3. Neutrophil Polarization and Function in I/R

**Figure 2:**
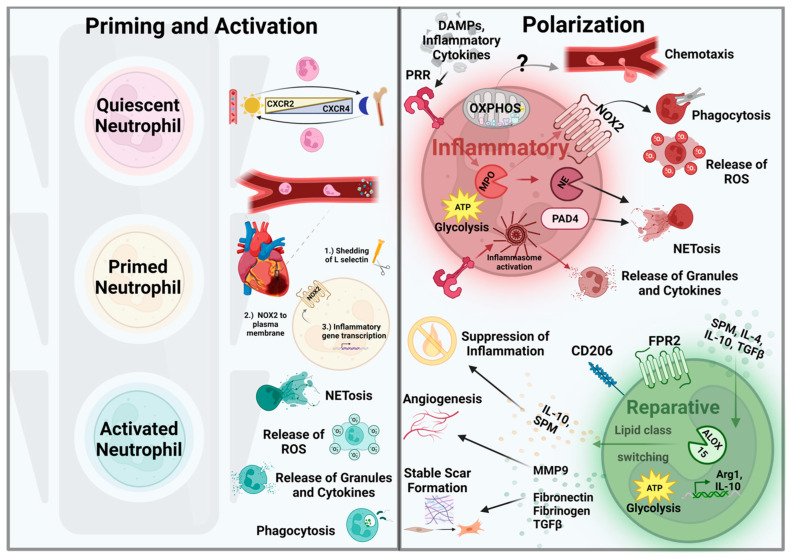
Neutrophil activation and polarization in myocardial I/R injury. Priming and activation: Under homeostatic conditions, quiescent neutrophils are released from the bone marrow into circulation and return to the bone marrow via reciprocal expression of CXCR2 and CXCR4, respectively, under circadian control. During injury, DAMPs prime neutrophils is in circulation, resulting in (1) the shedding of L-selectin, (2) the movement of NOX2 and integrins to the plasma membrane, and (3) the upregulation of inflammatory gene expression. At the site of injury, fully activated neutrophils elicit effector functions, including the release of ROS, NETs, granules/cytokines, and phagocytosis of pathogens, or in the case of I/R, cellular debris. Polarization: During I/R, DAMPs activate PRRs and promote neutrophil polarization toward an inflammatory phenotype. PRR signaling stimulates activation of the inflammasome and release of granules and inflammatory cytokines. Myeloperoxidase (MPO) is also activated, which in turn upregulates NOX2 and NE function, increasing ROS generation both within phagosomes and extracellularly. NE and PAD4 mediate the formation and release of neutrophil extracellular traps (NETs), which contribute to microvascular dysfunction during reperfusion (“no reflow”). During resolution, SPMs and anti-inflammatory cytokines signal through pro-resolution receptors (e.g., FPR2), to upregulate ALOX15 and facilitate a phenotypic switch toward repair. Reparative neutrophils undergo lipid class switching, increase expression of CD206, Arg1, and release resolving factors (e.g., IL-10), pro-angiogenic factors (e.g., MMP9), and profibrotic factors (e.g., TGFβ) that contribute to the resolution of inflammation and wound healing. Metabolically, neutrophils rely on glycolysis for most effector functions, but may require oxidative phosphorylation (OXPHOS) for proper chemotaxis.

Neutrophils rapidly infiltrate the heart within minutes of reperfusion, peaking at ~1 day post MI and lasting up to 3–4 days, with decreases detectable around day 5 and later in mouse models [39,41,50]. Recently, neutrophils were shown to polarize to heterogeneous phenotypes and subsets within the heart following MI in a time and context-dependent manner, similar to macrophages [39,41,46,48] (Figure 2). In the infarcted heart, neutrophils are activated by local inflammatory factors via PRR engagement, and polarize to a proinflammatory phenotype (sometimes referred to as “N1”), promoting their anti-microbial functions [39,43,52,53]. Polarization can also begin in the peripheral blood and the bone marrow in models of MI, a process that may involve neutrophil reverse transmigration to stimulate granulopoiesis and propagate inflammation [35,46]. Distinctively, proinflammatory “N1” neutrophils produce robust levels of ROS, release granules, and deploy chromatin NETs to neutralize perceived threats. Unlike most cells, neutrophils convert superoxide into secondary oxidants through the neutrophil-specific enzyme myeloperoxidase (MPO), the most abundant protein found in neutrophils, which are then used in microbial killing within the phagosome [54]. Importantly, release of extracellular granules and their specific components was shown to modulate the severity of the innate immune response by increasing chemotactic and inflammatory cytokines [55,56]. The generation of ROS and activation of NOX2 and MPO during phagosome formation also trigger the release and activation of neutrophil elastase (NE), which degrades the nuclear membrane of neutrophils and allows for formation and expulsion of extracellular traps. NETs can also be activated by citrullination of histones via protein arginine deiminase 4 (PAD4), which decondenses neutrophil chromatin and allows for expulsion. Decondensed chromatin released from the nuclear membrane adsorbs granule components and is released extracellularly to trap and prevent the spreading of pathogens [55].

Collectively, these proinflammatory neutrophil functions contribute to I/R injury in several ways. First, neutrophils were shown to infiltrate the area at risk during ischemia, and reperfusion accelerates infiltration and increases neutrophil numbers [39,43,52]. ROS production, NET release, and excessive degranulation from neutrophils increase cardiomyocyte death in the border zone and expand the infarct after I/R [16,39,44,48,57,58]. Second, neutrophil infiltration and NETs were shown to occlude the microvasculature of the myocardium, resulting in no-reflow reperfusion injury and additional cardiomyocyte loss [39,43,52,59]. Third, neutrophils secrete inflammatory cytokines/chemokines that stimulate monocyte and macrophage recruitment and proinflammatory differentiation to exacerbate injury [4,10,19,40,60]. For these reasons, the longstanding paradigm held that neutrophils primarily worsen cardiac injury. Indeed, inhibition of neutrophil mobilization and infiltration by disrupting adhesion molecules [61], as well as genetic inhibition of NETosis (*PAD4^-/-^* [62]), antimicrobial enzymes (neutrophil elastase, *ELANE^-/-^* [63]), or ROS production (NADPH Oxidase 2, *NOX2^-/-^* [64]) all demonstrated reduced infarct size in preclinical mouse MI models [18,42,57,61,62,63,65,66]. Intriguingly, however, global neutrophil depletion in mice subjected to MI resulted in worsened fibrotic remodeling and further impaired cardiac function, indicating a beneficial role for endogenous neutrophils in mediating cardiac wound healing [53].

As neutrophils are critical for the clearance of necrotic cell debris, a step that is instrumental for the initiation of resolution and repair, this may explain in part the accentuated pathology observed following antibody-mediated neutrophil depletion [39,53,67]. Consistently, recent work demonstrated that neutrophils exhibit significant plasticity and likely exist on a polarization spectrum not unlike the macrophage. Following the initial MI-induced inflammatory phase, neutrophils appear to play an important role in the resolution and cardiac repair responses by polarizing toward a reparative (“N2”) phenotype during the later wound healing phase [39,41,48,50,53,60,67,68,69]. Phenotypic modulation in neutrophils can be regulated, at least in part, by specialized pro-resolving lipid mediators (SPMs) [60,69,70]. During I/R, prostaglandins, leukotrienes, and omega-3 and -6 fatty acids accumulate within the damaged myocardium, upregulating the expression of arachidonate 15-lipoxygenase (ALOX15), and promoting the conversion to lipoxins and resolvins. These cardiac SPMs signal via the formyl peptide receptor 2 (FPR2) in both neutrophils and macrophages to suppress neutrophil recruitment and enhance efferocytosis and secretion of anti-inflammatory cytokines in macrophages [60,69,71]. Neutrophils that infiltrate the infarcted heart ~3–4 days post MI enter an environment of clearance, and polarize toward a resolution/reparative (“N2”) phenotype [39,41,50,60,68]. These reparative neutrophils respond to anti-inflammatory factors such as IL-4, IL-10, TGFβ, VEGF, and SPMs, and upregulate resolution-associated molecules Arg1 and IL-10, as well as release resolving lipid mediators lipoxin A4 and resolvin D1, similar to reparative macrophages [39,41,48,50,53,60,67,68,69,70]. Neutrophils also promote resolution through their regulated death [41,53,72]. Following MI, apoptotic neutrophils direct “M2-like” resolving macrophage polarization via macrophage efferocytosis of NGAL (a component of neutrophil granules) and resolvin D1, eliciting the upregulation of MerTK and enhanced phagocytotic capacitance [41,48,53,67,73]. Interestingly, neutrophils also contribute to cardiac repair by producing matrix proteins needed in fibrotic scar formation, such as fibronectin and fibrinogen, and can promote angiogenic responses via secretion of MMP9 [67,74]. These characteristics highlight the pleiotropic role of neutrophils within the myocardium during I/R injury.

### 3.4. Neutrophil Metabolism

Neutrophils can utilize a variety of energetic substrates depending on their environment, an important feature due to the broad range of oxygen and glucose concentrations experienced during cardiac I/R [3,10,16,75]. The impact of metabolic status on neutrophil function is therefore of great interest, yet studies directly examining this interaction remain limited [76]. In hypoxic environments, neutrophils favor glycolysis to generate ATP needed not only for survival, but also for NET formation and ROS production [77,78]. Studies also demonstrated that activated neutrophils upregulate OXPHOS genes and rely upon mitochondrial respiration to generate sufficient ATP necessary for cell migration and chemotaxis, a process shown to be mediated by mTORC1/2 signaling [79,80,81]. However, evidence linking electron transport chain function to neutrophil migration largely derives from bacterial infection studies, and whether this mechanism occurs during sterile inflammation, e.g., I/R, or if additional neutrophil effector functions are dependent upon altered metabolic status requires further investigation. Taken together, neutrophil polarization to inflammatory or reparative phenotypes is a spatially and temporally regulated mechanism during I/R, and their functional heterogeneity modulates the extent of myocardial injury and wound healing. As technological advances allow for more nuanced evaluation of neutrophil transcriptional, functional, and metabolic heterogeneity, more work is required to determine if and how these processes may integrate to regulate cardiac inflammation and injury caused by I/R.

## 4. Macrophages

### 4.1. Cardiac-Resident Macrophages

**Figure 3:**
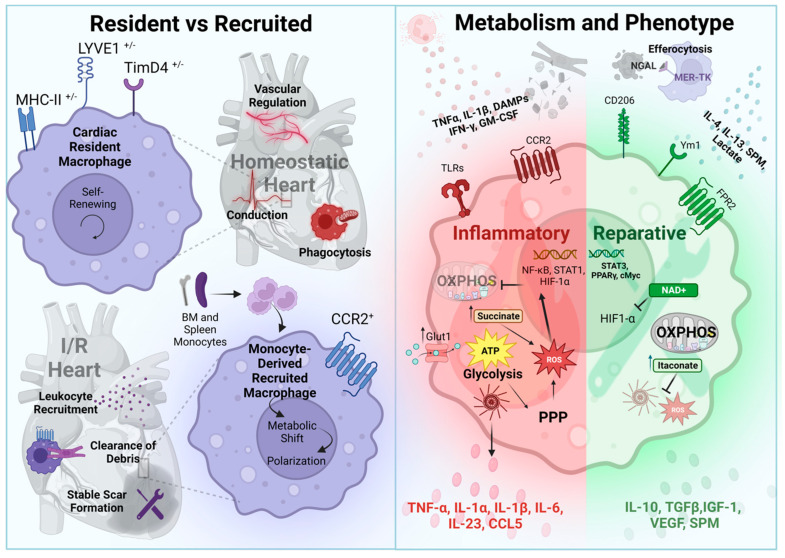
Macrophage regulation and function during myocardial I/R injury. Resident versus recruited: In the developing and homeostatic heart, self-renewing resident macrophages (CCR2^-)^ contribute to the formation and maintenance of the vasculature, electrical conduction, and phagocytosis of dysfunctional organelles in the myocardium. These macrophages can be subclassified by the expression of MHCII, LYVE1, and TimD4 on their cell surface. In response to injury, monocyte-derived macrophages (CCR2^+^) are recruited to the heart, where they further leukocyte infiltration during inflammation, clear dead cells and debris, and modulate stable scar formation during repair. Polarization and metabolism: DAMPs and inflammatory cytokines polarize recruited monocytes into inflammatory macrophages through activation of PRRs (e.g., TLR2/4). Upregulation of inflammatory genes (HIF-1α) and glucose transporters (Glut1) enhance glycolysis and suppress oxidative phosphorylation (OXPHOS). This results in the accumulation of succinate and activation of the pentose phosphate pathway (PPP), increasing ROS production. Activation of the inflammasome releases inflammatory and chemoattractant molecules, furthering inflammation. MerTK-mediated efferocytosis of dead cells, including neutrophils containing NGAL, as well as the binding of anti-inflammatory cytokines, initiates a phenotypic switch in the macrophage toward reparative function. Enhanced OXPHOS and the subsequent increase in itaconate and NAD^+^ suppresses inflammatory gene expression (HIF-1α) and effector functions (ROS, inflammasome). Reparative macrophages express more CD206, YM-1, and FRP2, and release mediators that contribute to revascularization and stable scar formation.

Macrophages are the most abundant leukocyte found in the heart and play a critical role in cardiac development, homeostasis, and injury responses [10,13,82,83,84,85,86,87,88,89]. Recent advances in sequencing and lineage tracing technologies demonstrated cardiac macrophage heterogeneity at steady state and following injury, revealing distinct ontologies, regenerative capacitance, surface markers, and spatial and functional niches [4,13,82,84,85,86,87,90]. At a basic level, macrophages can be classified as resident or recruited cells [13,82,84,85,86,87,91]. Cardiac-resident macrophages originate from the yolk sac and fetal liver during development, whereas recruited macrophages are derived from peripheral monocytes and are generally denoted as CCR2^+^ [13,82,84,85,86,87,91]. CCR2^−^ resident macrophages are maintained through self-renewal and can be further classified by their relative expression of MHCII, TimD4, and Lyve1. These tissue-resident macrophages perform homeostatic functions within the heart. They are important for the proper patterning of coronary and lymphatic vessels during development, they facilitate cardiomyocyte conduction, phagocytosis of dysfunctional ejected mitochondria (exophers) and cellular debris, regulate angiogenesis, and provide defense against pathogens [13,82,84,85,86,87,90,91,92,93]. Importantly, humans and mice demonstrate significant conservation in cardiac-resident macrophage subset markers and function [88]. Following acute MI, some resident macrophages die; however, the surviving subset of these cells responds to injury and proliferates (far fewer perish if reperfusion is provided [94]). Importantly, cardiac-resident macrophages also negatively regulate the recruitment of monocytes to the heart, thereby antagonizing the inflammatory process [13,82,85,90,95] (Figure 3). Indeed, depletion of cardiac-resident macrophages through genetic targeting approaches worsened pathological remodeling in response to MI, suggesting cardioprotective effects of these cells in response to heart injury [90,95]. Thus, the resident macrophages within the myocardium are a unique cell subpopulation with distinct functional states that are essential to cardiac development, homeostasis, and stress responses.

### 4.2. Cardiac Recruited Macrophages

In response to acute MI, there is a rapid and robust increase in circulating pro-inflammatory (CCR2^+^) monocytes derived from the bone marrow and spleen [4,10,13,82,84,85,86,87,90,95]. Studies in mice demonstrated that monocytes arriving soon after MI differentiate into pro-inflammatory macrophages, whereas monocytes recruited later in the immune response differentiate into macrophages that favor reparative phenotypes [4,10,13,82,86,87,90,95]. Recruited CCR2^+^ macrophages are mobilized to the injured heart during both reperfused (I/R) and non-reperfused MI models; however this process is accelerated by reperfusion, with recruited macrophages present in the heart by 24 h post I/R, peaking ~3 days, and declining by days 7–14 [4,13,84,86,87]. During the initial inflammatory phase (days 1–4), CCR2^+^ monocytes, stimulated by GM-CSF, TNFα, IL-1β, and IFNγ, differentiate into inflammatory CCR2^+^ macrophages [87,96]. These inflammatory macrophages clear debris and dead cells at the injury, which limits secondary necrosis and prevents cardiac rupture. Macrophage phagocytosis/efferocytosis is a carefully coordinated combination of “find me” (e.g., DAMPs) and “eat me” (e.g., phosphatidyl serine) signals that bind phagocytic receptors on the macrophage, such as Tyro3, Axl, MerTK, and CD36 [4,10,84,86,87]. However, recruited CCR2^+^ macrophages also upregulate several key transcription factors (e.g., NF-κB, STAT1, and HIF1α) to produce cytokines/chemokines that further propagate monocyte recruitment and inflammation [4,10,84,86,87]. Importantly, this positive feedback is thought to contribute to the collateral loss of cardiomyocytes and expansion of the infarct, which further worsens adverse cardiac remodeling and dysfunction. Indeed, targeting CCR2 in circulating monocytes and preventing their recruitment to the injured heart damped the inflammatory response and improved outcomes following MI [97].

### 4.3. Macrophages in Resolution

As the inflammatory process progresses, the efferocytosis of dead neutrophils, cardiomyocytes, and other parenchymal cells, as well as contribution from the adaptive immune system, induce a phenotypic switch and favor a reparative macrophage state, formerly referred to as alternatively activated macrophages [4,84,86,87,96,98,99]. Reparative macrophages can be induced by IL-4 and IL-13, among other factors, which leads to the upregulation of molecules important for wound healing (e.g., Arg1, CD206, VEGF, IGF-1, and YM1) via the activation of transcription factors MYC, PPARγ, STAT3, and others [4,84,86,87,96]. In addition, monocyte-derived macrophages that are recruited during the later phase of MI downregulate Ly6C expression and polarize to a reparative phenotype (~4–7 days post I/R) [4,84,86,87,96]. The secretion of pro-resolving mediators from these macrophages, such as IL-4, IGF-1, and TGFβ, act in a paracrine fashion to further propagate reparative macrophage polarization, as well as elicit cardiac fibroblast differentiation into myofibroblasts, thereby mediating extracellular matrix remodeling and subsequent scar formation after MI [4,84,86,87,96]. Reparative macrophages also produce angiogenic and lymphogenic factors, such as VEGFA and VEGFC, respectively, to promote revascularization and repair of the damaged myocardium after infarction [92].

### 4.4. Macrophage Metabolism and Functional Regulation

As alluded to above, the infarcted heart is a hostile environment with extreme ranges of available oxygen, nutrients, and metabolites. Inflammatory monocytes mobilized acutely during MI are highly dependent upon glycolysis, and increased glycolytic flux in monocytes is correlated with increased secretion of inflammatory cytokines, increased infarct size, and decreased systolic function in human MI patients [100,101]. Glucose uptake and greater dependence on glycolysis relative to oxidative respiration provide the energy necessary for proliferation, inflammatory cytokine production, generation of ROS, and adhesion and transmigration of monocytes into the heart following MI. This reliance on glycolysis continues as inflammatory monocytes differentiate into CCR2^+^ macrophages. Hypoxic conditions that result from ischemia activate macrophage HIF-1α, which upregulates glucose transporters (e.g., Glut1), as well as glycolytic enzymes (e.g., PDK1, hexokinase, 6-PFK), all of which favor glycolysis [13,84,87,95,100,101,102]. This also activates the pentose phosphate pathway (PPP), which provides NADPH needed for ROS generation through NADPH oxidase. Moreover, HIF-1α positively regulates the production of IL-1β and stimulates the proteolysis and inhibition of the phagocytic receptor MerTK, which is needed for reparative macrophage function [103]. Preventing HIF-1α-mediated glycolysis attenuated these responses [104,105]. Inflammatory CCR2^+^ macrophages also exhibit impaired TCA cycle and oxidative phosphorylation, which causes the accumulation of TCA metabolites that can influence cell behavior. In particular, succinate is oxidized during reperfusion, leading to the reversal of electron transport chain complex I and increased ROS, further stabilizing HIF-1α and exacerbating inflammatory conditions [106,107].

In contrast to proinflammatory CCR2^+^ cardiac macrophages, reparative macrophages are associated with mitochondrial OXPHOS, although this is an oversimplification and both oxidative respiration and glycolysis can influence pro- and anti-inflammatory mechanisms [108]. Following MI, genes related to mitochondrial respiration are upregulated and several mitochondrial-related metabolic intermediates were shown to promote macrophage-reparative functions. For example, TCA cycle-derived itaconate antagonizes the oxidation of succinate and blunts complex I-generated ROS to suppress inflammation. Itaconate was also shown to attenuate inflammasome function, and itaconate supplementation in vivo was demonstrated to reduce infarct size and subsequent adverse cardiac remodeling [109,110,111]. The TCA cycle also generates NADH, which when oxidized, produces NAD^+^, a potent anti-inflammatory metabolite. Indeed, NAD^+^ levels are reduced in heart failure and supplementation of NAD^+^ or precursors that increase NAD^+^ levels were shown to protect against cardiac I/R injury and increase reparative macrophage populations [112,113,114,115]. NAD^+^ is inhibitory against HIF-1α and can promote its degradation, thereby favoring macrophage reparative polarization [116].

In response to efferocytosis, macrophages direct excess lipids to mitochondria for increased fatty acid oxidation and mitochondrial respiration, which activates PGC-1β and elevates NAD^+^ levels, leading to a reparative phenotype [117]. Consistent with these findings, disruption of the electron transport chain complex I function in the myeloid compartment resulted in an augmented inflammatory response and worsened cardiac injury after MI [118]. The process of removing dying cells also induces the synthesis of IL-10, another pro-reparative cytokine that plays an important role in resolution and wound healing [73]. Efferocytosis can also stimulate glycolysis and cause increased lactate in the macrophage. Interestingly, lactate can be secreted to elicit paracrine effects in neighboring cells that promote macrophage anti-inflammatory polarization [119]. Together, these findings support the concept that selective activation of metabolic pathways and the relative enrichment of individual metabolites have the capacity to alter macrophage polarization and function, thus impacting cardiac outcomes following injury. How this regulation might be impacted by cardiovascular comorbidities, such as aging and obesity/metabolic syndrome, and whether macrophage metabolism can be harnessed for therapeutic benefit, remains unclear and warrants future investigation.

## 5. Dendritic Cells

**Figure 4:**
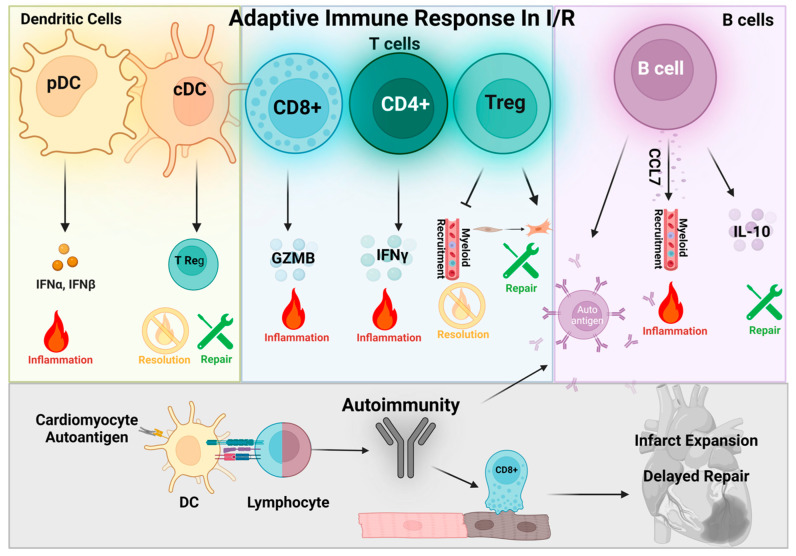
The adaptive immune response during myocardial I/R injury. Dendritic cells: Cardiac resident dendritic cells (DCs) can be subclassified into plasmacytoid (pDC) and conventional (cDC) subtypes. During I/R, pDCs increase production and release type I interferons and contribute to the pro-inflammatory response. Conversely, cDCs were shown to facilitate the recruitment of Tregs and may favor resolution and repair processes. T cells: T cells are both resident and recruited to the heart during I/R, and can be classified into three main subtypes: cytotoxic T (CD8+), T helper (CD4+), and T regulatory (Treg). CD8+ cells release granzyme B (GZMB), promoting cardiomyocyte apoptosis and inflammation, while CD4+ cells release interferon gamma, which also promotes inflammation. In contrast, Tregs promote resolution by suppressing myeloid recruitment and stimulating fibroblast activation and repair after I/R. B cells: Similar to T cells, B cells are both resident and recruited to the heart after I/R. B cell activation and contribution to I/R injury remains unclear and likely has pleiotropic effects. B cells can positively regulate CCL7, which promotes myeloid cell recruitment and cardiac injury, yet B cells also produce IL-10 that can stimulate resolution and repair following I/R. Autoimmunity: The massive loss of cardiomyocytes elicited to I/R can trigger autoimmune responses in the heart. Resident DCs can present myocardial debris as autoantigens to T and B cells. B cells differentiate into plasma cells (PCs) and produce autoantibodies against the heart, while CD8+ T cells target healthy cardiomyocytes, contributing to infarct expansion and delayed repair.

Cardiac resident myeloid cells also include dendritic cells (DCs), which coordinate both the innate and adaptive immune responses via antigen presentation and cytokine release [88,120]. Similar to macrophages, DCs are heterogeneous and consist of two major subsets within the heart, conventional (cDC) and plasmacytoid (pDC), which can be selectively targeted in vivo [120,121,122,123]. In the context of acute MI, both cDCs and pDCs expand and activate within the myocardium, and global depletion of DCs worsened MI-induced remodeling and dysfunction, suggesting that a component of DC function contributes to wound healing [122]. However, the selective individual inhibition of either subtype was shown to be cardioprotective, indicating a deleterious role for DCs, although particular subtype contributions may differ in the absence of reperfusion [120,121,123]. pDCs were shown to release type I interferons early after I/R, and their depletion afforded cardiac benefit [120]. cDCs contribute to pathological remodeling post MI, presumably through alterations in macrophage and Treg recruitment, as demonstrated by selective cDC depletion [121,122,124]. Importantly, dendritic cells specialize in antigen presentation and further modulate MI injury through auto-reactive T cell activation [125,126,127]. For example, necrotic cardiomyocytes release α-myosin heavy chain, which is presented to T cells by DCs in the mediastinal lymph nodes or circulating blood, resulting in heart-specific autoimmune responses that contribute to prolonged inflammation and exacerbate I/R injury [125,128] (Figure 4).

During steady state, DC metabolism relies mainly on fatty acid oxidation and OXPHOS; however, following TLR-mediated activation, DC metabolism shifts to glycolysis [129,130]. This transition promotes inflammatory cytokine secretion and antigen presentation [129]. Intriguingly, DC subsets display distinct metabolic profiles upon activation, as cDCs favor glycolysis and pDCs favor oxidative phosphorylation [131]. How these differences in energetics might modulate inflammatory behaviors in DC subsets, and whether this contributes to functional differences in MI injury and remodeling remains largely unknown. However, given the implied importance of DCs in heart disease after injury, further elucidation of mechanisms underlying their activity and interactions with other immune cell types may uncover novel therapeutic targets to improve cardiac outcomes after I/R.

## 6. T Cells

T cells are lymphoid derived and make up ~25% of the non-myeloid resident leukocytes in the myocardium [13,100]. T cells are broadly categorized as CD4+ T helper and CD8+ cytotoxic T, with subset classification into multiple effectors, including regulatory T cells (Tregs) [127]. Similar numbers of CD4+ and CD8+ T cells were observed in the healthy myocardium [132], although their relative contribution to cardiac homeostasis remains elusive. Following acute MI, a subset of T cells infiltrates the heart prior to antigen activation [133], and contributes to injury by secreting inflammatory cytokines, enhancing leukocyte recruitment, and expanding the infarct [4,134,135]. Interestingly, antibody depletion of CD4+, but not CD8+, T cells immediately after I/R improved cardiac outcomes, which was attributed to suppressed interferon gamma secretion and reduced neutrophil recruitment [135]. However, in non-reperfused MI, CD8+ antibody depletion was shown to be cardioprotective by suppressing granzyme B-mediated cardiomyocyte apoptosis [136]. As mentioned above, dendritic cell-mediated antigen presentation to T cells elicits activation of the adaptive immune response and initiates autoimmunity against the heart, prolonging the inflammatory response and causing additional cardiac damage [4,127,137,138,139]. On the other hand, Tregs demonstrated cardioprotective effects that include restraining CD4+ and CD8+ inflammatory effector functions during acute MI and the subsequent remodeling process [137,140,141,142]. Tregs, similar to other T cells, accumulate in the heart post MI, yet have distinct transcriptional profiles acquired by activation in the inflamed myocardium that favor a reparative phenotype. After ischemic insult, Tregs were shown to suppress myeloid cell recruitment, as Treg depletion increased inflammatory monocytes and neutrophils in the infarct zone [143]. Upon activation by DCs, Tregs also facilitate wound healing and scar formation, and prevent rupture, via the activation of resident cardiac fibroblasts and increased reparative macrophage polarization after MI [142,143,144]. Furthermore, depletion of FoxP3^+^ Tregs enhanced cardiac autoimmunity after I/R, indicating that Tregs normally protect the heart by actively suppressing autoreactivity [141]. Finally, Tregs may also provide cardioprotection during ischemic heart injury by promoting cardiomyocyte proliferation, demonstrated by Treg supplementation post MI [145,146] (Figure 4).

T cell metabolism and potential functional implications, in the context of ischemic heart disease, is currently an active area of investigation, and thus far appears to play an important regulatory role in T cell differentiation and effector functions [100]. Similar to macrophages, an upregulation of glucose transporters and a metabolic preference for glycolysis promotes CD4+ T cell expansion and production of interferon gamma, as well as the expression of granzyme B in CD8+ T cells [147,148]. Conversely, Tregs express lower levels of Glut1 and exhibit greater fatty acid oxidative and mitochondrial respiration, suggesting that reduced reliance on glycolysis may underly the anti-inflammatory function in these cells [100,148,149]. Given the distinct transcriptional and metabolic profiles exhibited by cardiac T cells during ischemic injury, it will be important to determine whether metabolic pathway preferences modulate T cell differentiation, function, and potentiate T cell-mediated autoimmunity following I/R.

## 7. B Cells

B cells are one of the most abundant leukocyte populations in the healthy myocardium, yet our understanding of B cell function in the heart, during both homeostatic conditions and following injury, remains relatively limited [127,132,150]. For example, the role of cardiac resident B cells in maintaining cardiac homeostasis is largely unexplored; however, prior work suggests that B cells can regulate MHCII expression on resident macrophages in the heart [151]. B cell numbers expand after MI [127,150,152,153] and are thought to play a role in acute injury and inflammation, as well as autoimmune responses during chronic remodeling [127,150,152,153]. Depletion studies demonstrated that the B cell inhibition attenuated inflammatory myeloid cell infiltration and subsequent pathological cardiac remodeling due to repressed CCL7 secretion after MI [153]. Additionally, the production of autoantibodies by B cells after I/R was shown to contribute to enhanced cardiomyocyte loss and neutrophil infiltration [154]. In contrast, studies in mice that overproduce B cells also demonstrated preserved cardiac function post MI [155], and adipose-derived pericardial B cells were shown to secrete cardioprotective IL-10 [156]. These findings demonstrate the pleiotropic nature of these lymphocytes and highlight the need to further define the complex mechanisms underlying the role of B cell function during cardiac injury (Figure 4). Recent work found that metabolic status can influence B cell function. For example, activation of the B cell receptor increases glycolysis, and subsequent autoantibody and cytokine production in B cells appears to be energetically supported by glycolysis [100,157], whereas production of IL-10 relies primarily on fatty acid oxidation [158]. Moreover, differentiated B cells (plasma cells) were shown to favor fatty acid oxidation relative to glucose utilization for enhanced survival; however, whether this mechanism is conserved in ischemic heart disease remains unknown [159].

## 8. Translational Potential of Targeting Inflammation during I/R Injury

Inflammation plays a fundamental role in modulating I/R-induced injury and wound healing and is therefore of great interest for potential therapeutic intervention. Multiple lines of reasoning provide a compelling rationale for modulating post-I/R inflammatory cascades for cardiac benefit. These include reduced leukocyte-mediated death of vulnerable cardiomyocytes at the infarct border, reduced ECM remodeling and strengthened scar formation, enhanced angiogenic effects, potential reduction in arrhythmogenic incidence, and protection against future coronary events and recurrent MI in humans. Moreover, some standard treatments for MI patients, e.g., β-adrenergic receptor antagonists, may have inherent anti-inflammatory functions that contribute to their therapeutic efficacy [160]. Many preclinical studies leveraged our understanding to target inflammatory processes, and showed promise for ameliorating infarction and subsequent pathological remodeling and cardiac dysfunction (Table 1). However, interventions aimed at depleting entire populations of cells (e.g., neutrophils or macrophages [53,161]) or employing broad inhibitors of inflammatory processes had limited success–probably because these cells and processes have pleiotropic effects in the inflamed myocardium. Implementing strategies to effectively target inflammation clinically remains challenging due to multiple translational barriers and will likely require enhanced precision and better understanding of patient heterogeneity.

### 8.1. Broad Approaches to Inflammatory Inhibition

Glucocorticoids have wide-ranging effects on many cell types, and were shown to both promote and antagonize inflammatory function depending on the cellular target and status of the injury environment [179]. Early studies noted a benefit of glucocorticoid administration for reducing acute MI injury in preclinical animal models; however, subsequent studies reported mixed results, in some cases worsening cardiac outcomes [180,181,182]. This may be the result of the ability of glucocorticoids to signal via both glucocorticoid receptors (GRs) and mineralocorticoid receptors (MRs), thereby affecting a broad range of cell types to elicit systemic effects [183]. Glucocorticoids were also shown to signal through MRs to enhance inflammatory mediators [184,185], and high dose treatment led to dysregulation of macrophage clearance of cellular debris from the infarct site and impaired fibroblast function, leading to compromised scar formation [182]. Importantly, results from the clinical application of glucocorticoids for MI patients did not demonstrate convincing effectiveness overall, and several studies raised safety concerns, making this broad approach to limit inflammation largely unattractive [186].

Nonsteroidal anti-inflammatory drugs (NSAIDs) also act to broadly repress inflammatory responses through the inhibition of COX enzymes. Similar to findings employing glucocorticoids, NSAID administration showed mixed results in preclinical studies, with some reports of protection against MI-induced cardiomyocyte death and adverse remodeling, and others reporting worsening cardiac dysfunction, scar thinning, and a protective effect of COX-2 during I/R injury [187,188,189,190,191]. Attempts to translate this approach to patients were largely unsuccessful, with no clear benefit for MI reduction observed, and an increased risk of death and recurrent MI [192,193]. These adverse outcomes may be due to local and/or systemic effects including altered blood pressure, atherogenic predisposition, attenuated repair processes, or enhancement of arrhythmogenic events [194].

Cyclosporine A (CsA) is an immunosuppressant that targets and inhibits the function of cyclophilin D, thereby preventing the opening of the mitochondrial PTP and cell death [195]. In preclinical animal models of I/R, CsA was somewhat effective at reducing injury and potentially cardiac inflammation, although results were inconsistent [196,197]. Early pilot results in MI patients administered CsA showed promise [198]; however, follow-up investigation did not find a significant benefit when combined with PCI therapy [199].

Ischemic conditioning (IC) is a mechanical intervention for producing multiple cycles of non-lethal ischemia followed by reperfusion. IC can be applied directly to the coronary artery (in the case of MI) to elicit endogenous cardiac benefit, or to vasculature/organs remote from the heart, referred to as remote ischemic conditioning (RIC). Since its discovery more than three decades ago [200], IC has been shown to effectively protect the myocardium against I/R injury in a variety of preclinical models [201]. The underlying mechanisms that afford cardioprotection are thought to consist of the reperfusion injury salvage kinase (RISK) pathway, the survivor activating factor enhancement (SAFE) pathway, and the PKC-NO-PKG pathway [202]. Recent work also implicated RIC as a modulator of inflammatory signaling in response to I/R, which may facilitate additional myocardial protection. Preclinical evidence from studies in mice, rats, and rabbits demonstrate attenuated pro-inflammatory cytokines (e.g., TNFα, IL-1β, and IL-6) and inflammatory mediators (e.g., TLR4, HMGB1, and ICAM-1) when animals are subjected to RIC compared to I/R without treatment [203,204,205,206,207]. Unfortunately, the robust protective effect of IC is not consistently observed in MI patients, and large randomized controlled trials have been largely inconclusive, with no improvement in clinical outcomes after one year [208,209,210]. Discrepancies in clinical observations could be due to limitations inherent to animal models of acute MI [211], as well as our limited understanding of mechanisms that convey the cardiac benefits of RIC. In this regard, results from the clinical administration of RIC in coronary artery bypass graft (CABG) patients did not demonstrate a clear effect on inflammatory mediators, and whether this approach modulates inflammation caused by I/R in humans requires additional investigation [212,213,214,215].

### 8.2. Focused Targeting of Inflammatory Cells

#### 8.2.1. The Complement Pathway

The complement cascade is activated by DAMPs following I/R and plays a role in modulating both the extent of injury and the inflammatory response in the infarcted heart [26,216]. Inhibition of the complement pathway in both small and large animal preclinical studies demonstrated cardioprotection against acute MI [163,164,217]. Attempts to translate these findings to the clinic, however, have not been successful. For example, treatment with pexelizumab to target complement inhibition in STEMI patients did not improve infarct size, mortality, or heart failure development [218,219,220].

#### 8.2.2. Targeting Immune Cell Recruitment and Adhesion

As discussed above, there are several mechanisms that modulate the recruitment of neutrophils, monocytes/macrophages, and lymphocytes to the injured myocardium post I/R. Attempts have been made to target these mediators and disrupt normal migration, adhesion, and extravasation, in an effort to reduce inflammatory damage and increase cardioprotection. Inhibition of certain chemokine function demonstrated limited cardiac benefit in small animal studies. Blockade of CCL2 or CCL5 improved post-MI remodeling and cardiac function, presumably through attenuation of proinflammatory cell recruitment to the injured heart [221,222]. Similar results were reported using an RNAi approach to deplete CCR2, which impaired recruitment of inflammatory monocytes and reduced infarct size in mice [223]. Of course, manipulating CC chemokines may also interfere with recruitment of immune cells that provide salutary effects. Indeed, CCR5 knockout mice demonstrated worsened cardiac remodeling after I/R [224], which may be a result of impaired inflammatory resolution, and highlights the delicate balance that should be maintained to limit injury and maximize wound healing.

Integrins and selectins are important for leukocyte adhesion to, and extravasation through, the endothelium following I/R. Strategies that leveraged antibodies to neutralize these mediators demonstrated cardioprotection and reduced infarct size post I/R in preclinical models [225,226,227]. Moreover, concomitant depletion of multiple cell adhesion molecules using nanoparticle-mediated administration of siRNA was effective at improving cardiac function following MI in mice [228], indicating the potential to target adhesion therapeutically. Despite these findings, however, clinical trial results of anti-adhesion molecule treatments (anti-CD11/CD18) in MI patients have been underwhelming and did not afford infarct size reduction [229,230,231]. Administration of the P-selectin antagonist inclacumab for MI showed more promise and may provide modest protection against cardiac damage; however, no difference in adverse events was observed between treatment regimens [232].

#### 8.2.3. Targeting Immune Cell Function and Inflammatory Mediators

Recent preclinical and clinical data suggest that targeting the bioactive molecules that are produced by inflammatory immune cells can provide benefit to the injured heart. The IL-1β/IL-1R pathway has emerged as an intriguing therapeutic target for treatment of I/R injury. Studies in mice demonstrated that either inhibiting IL-1β activity via treatment with anti-IL-1β neutralizing antibodies, or preventing IL-1R signaling using the receptor antagonist anakinra, can attenuate post-MI remodeling and improve heart function [233,234]. Importantly, this benefit may extend to patients, as administration of anakinra demonstrated significant reductions in C-reactive protein (CRP), death, and hospitalization [235,236,237,238]. Results from the CANTOS trial, which leveraged IL-1β neutralization by canakinumab in patients with previous MI, are also positive and demonstrate reduced markers of inflammation and reduced cardiovascular events and hospitalization [239,240,241]. However, risk of infection was increased, and the long-term safety profile of this approach requires continued study. The production of mature IL-1β is dependent upon the inflammasome, a multi-protein complex that is highly expressed in leukocytes, as well as in fibroblasts and to a lesser extent, cardiomyocytes [242]. Small molecule inhibitors that target inflammasome function have been used in preclinical MI models and shown to protect against I/R injury [170,171,172,233,243], yet examination of the therapeutic potential of this approach in patients has only recently begun [244] and warrants further investigation.

Colchicine is an anti-inflammatory drug that was shown to dampen inflammation in patients with MI [245]. Colchicine disrupts microtubule networks and negatively regulates the migration and infiltration of neutrophils following injury, which is thought to be a prominent mechanism providing immunosuppression [246]. Additional evidence implicates colchicine as an inhibitor of inflammasome function, which may also contribute to the attenuation of inflammatory burden [247]. Pilot evaluation of colchicine for treatment of acute MI in patients found a reduction in cardiac injury [248]. Results from the subsequent larger COLCOT study, which also administered colchicine to patients after acute MI, demonstrated a reduction in serious adverse events [245]. These data also indicate that colchicine may delay progression of heart failure; however, no reduction in infarct size was observed in this patient population, suggesting colchicine may provide benefit by modulating maladaptive remodeling post I/R.

Inhibitory targeting of IL-6, a prominent pro-inflammatory cytokine involved in reperfusion injury and heart failure [249], is an active area of research for the treatment of acute MI. Preclinical studies have demonstrated that blocking IL-6 signaling affords cardioprotection against I/R injury, including infarct reduction and preservation of cardiac function [250]. These promising results, however, are yet to be fully realized clinically. Administration of the IL-6 receptor antagonist tocilizumab reduced markers of cardiac injury in patients with acute MI, including troponin and CRP, as well as leukocyte counts, yet failed to reduce infarct size [251,252,253]. While these initial studies demonstrate promise for this approach, additional evaluation is needed in larger patient cohorts.

## 9. Conclusions and Future Perspectives

Advancements in treatments have significantly improved the survival of patients experiencing acute MI, yet there remains a great need for more effective therapies to further reduce cardiac ischemia and reperfusion injury and prevent adverse remodeling and accompanying heart failure. Inflammation plays a critical role in modulating cardiac injury and wound healing, and is therefore an attractive process for targeting novel therapies. However, the inflammatory response within the injured myocardium is complex, involving multiple cell types that exhibit pleiotropic effects and interact in multifaceted ways to dictate the timing and extent of damage and wound healing. Due to the intricate nature of these cellular and molecular responses, future studies will likely benefit from our growing detailed understanding of immune cell subpopulations during I/R injury and technological advances allowing for more precise targeting of therapeutic interventions for selective cell types and/or molecules at specific time points.

While the majority of interventions aim to suppress the pro-inflammatory aspects of cardiac inflammation after MI, it has also been implied that the ability to resolve inflammation, i.e., negatively regulate pro-inflammatory function, may be impaired in some patients and could contribute to pathogenesis of I/R injury and subsequent remodeling [254]. In line with this approach, preclinical studies have investigated the therapeutic potential of promoting reparative properties of immune cells post injury to confer cardiac benefit [255]. As mentioned above, SPMs signal through the FRP2 receptor to polarize macrophages and neutrophils toward a resolving phenotype and stimulate wound healing. A recent study found that treatment of rats with BMS-986235, a FRP2 agonist, increased phagocytosis and neutrophil clearance, and improved post-MI remodeling and heart function [256]. It is possible that future therapies will incorporate multiple aspects of immunomodulation in conjunction for added benefit, yet must also consider additional challenges involving inflammatory targeting for patients with MI. The timing of cellular actions and production of inflammatory mediators is critical, and interventions that disrupt early processes could unintentionally impact later responses and worsen outcomes. Therefore, further elucidating the time course of myocardial inflammation is likely to focus the effective window for therapeutic intervention. Moreover, patients are highly diverse and their susceptibility to inflammation and responsiveness to treatments can vary depending on age, gender, medical co-morbidities, genetic background, as well as other factors. In addition, preclinical work is performed largely in rodent models, which are typically young, healthy, and lack co-morbidities often present in humans. New treatments are usually not tested in combination with standard care regimens that patients with cardiovascular disease commonly rely on, which may also impact responses. Despite these challenges, results from some recent trials targeting inflammation for acute MI show promise and reaffirm the potential of manipulating the immune environment for myocardial salvage and improved patient prognosis.

## Figures and Tables

**Table 1:** Studies targeting inflammation in preclinical models of I/R. A representative list of relevant small animal studies that modulated inflammatory pathways and assessed cardiac injury and/or function following I/R. Findings from most preclinical studies suggest that attenuating pro-inflammatory responses generally provides cardiac benefit during I/R. ↓, reduced compared to control. ↑, increased compared to control.

I/R Model	Cell/Molecular Target	Animal Model/Intervention	Major Findings	Proposed Mechanism	Ref
Ischemia: 45 min Reperfusion: 1, 3, 5, and 7 days	Characterize overall immune response in the heart	Mice Flow cytometry	Reperfusion accelerated immune cell infiltration versus non-reperfused MI	Speculate early resolution of inflammatory response in the reperfused heart	[96]
Ischemia: 30 min Reperfusion: 24 h	TLR2 signaling	Mice TLR2^-/-^ global KO Administration of OPN-301 (TLR2 inhibitor)	↓ infarct size ↓ myeloid infiltration ↓ inflammation ↓ cardiomyocyte apoptosis ↑ cardiac function	Attenuated p38-MAPK and JNK signaling	[23]
Ischemia: 45 min Reperfusion: 3 days	TLR3 signaling	Mice TLR3^-/-^ global KO	↓ infarct size ↓ cardiomyocyte apoptosis ↓ myeloid infiltration ↑ cardiac function	Attenuated NF-κB and TNFα signaling. Reduced BAX/Bak signaling.	[28]
Ischemia: 30 min Reperfusion: 24 h 7 and 28 days	TLR4 signaling	Mice TLR4^-/-^ global KO Administration of TAK-242-NP (TLR4 inhibitor)	↓ infarct size ↓ myeloid infiltration ↓ inflammation ↓ pathological remodeling ↑ cardiac function	TLR4 inhibition at reperfusion suppressed CCR2-mediated inflammatory cell recruitment	[21]
Ischemia: 1 h Reperfusion: 24 h	TLR4 signaling	Mice TLR4 deficient strains	↓ infarct size ↓ inflammation	Attenuated neutrophil infiltration, reduced ROS, and reduced C3 complement	[18]
Ischemia: 30 min Reperfusion: 1 h (ex vivo in mice)	RAGE signaling	Mice: RAGE^-/-^ global KO Rats: Administration of soluble RAGE (sRAGE) decoys	↓ cardiac injury ↓ cGMP, nitrite/nitrate levels in myocardium ↑ Energy metabolism	Attenuated iNOS signaling, possibly due to decreased glycolysis and peroxynitrite formation	[25]
Ischemia: 30 min Reperfusion: 2 weeks	RAGE signaling	Mice Administration of soluble RAGE (sRAGE) recombinant protein	↑ cardiac function ↑ angiogenesis ↓ pathological remodeling ↓ endothelial apoptosis in myocardium	Increased angiogenesis via STAT3-mediated activation of VEGFR2 in myocardial endothelial cells	[162]
Ischemia: 30 min Reperfusion: 1 and 24 h 4 weeks	Complement cascade	Mice C5aR^-/-^ global KO	↓ infarct size ↓ leukocyte infiltration ↓ cardiomyocyte apoptosis ↑ cardiac function	Decreased neutrophil and T cell infiltration and related inflammation	[163]
Ischemia: 30 min Reperfusion: 4 h	Complement cascade	Rats Use of 18A, 16C (C5 neutralizing antibodies)	↓ infarct size ↓ cardiac injury ↓ myeloperoxidase ↓ cardiomyocyte apoptosis	Attenuated neutrophil infiltration and preserved C3b-related immunoprotection	[164]
Ischemia: 30 min Reperfusion: 24 h	NF-κB pathway	Mice p50^-/-^ global KO	↓ infarct size ↑ inflammation ↓ neutrophil infiltration	Suppressed adhesion of leukocytes	[165]
Ischemia: 30 min Reperfusion: 1 h (ex vivo) 24 h	NF-κB pathway	Mice p65 cardiac KO	↓ infarct size ↓ cardiomyocyte apoptosis ↑ cardiac function	Sustained intracellular calcium cycling, possibly through alterations in PLN	[166]
Ischemia: 30 min Reperfusion: 2 h	NF-κB pathway	Mice Administration of Bay 65-1942 (IKKβ inhibitor)	↓ infarct size ↓ cardiac injury ↓ inflammation ↑ cardiac function	Suppression of TNFα and IL-6	[167]
Ischemia: 30 min Reperfusion: 2 h	TNFα signaling	Mice TNFα^-/-^ global KO TNFα neutralizing antibodies	↓ arrhythmia ↓ infarct size ↓ inflammation ↑ cardiac function	Attenuated NF-κB activation Reduced neutrophil infiltration	[168]
Ischemia: 30 min Reperfusion: 24 and 48 h	NLRP3 Inflammasome	Mice ASC^-/-^ global KO Caspase-1^-/-^ global KO	↓ infarct size ↓ pathological remodeling ↓ myeloid infiltration ↓ inflammation ↑ cardiac function	Activation of the inflammasome in fibroblasts facilitates leukocyte infiltration.	[169]
Ischemia: 30, 75 min Reperfusion: 1, 3, 6, and 24 h	NLRP3 Inflammasome	Mice Administration of NLRP3 inhibitor (NLRP3inh)	↓ infarct size ↓ caspase-1 activity	Early inhibition of NLRP3 after reperfusion suppressed pyroptotic cell death	[170]
Ischemia: 30 min Reperfusion: 3, 24, and 48 h	NLRP3 Inflammasome	Mice Administration of NLRP3 siRNA or BAY 11-7028 (inflammasome inhibitor)	↓ myeloid infiltration ↓ cardiomyocyte apoptosis ↓ infarct size ↑ cardiac function	Suppression of ROS-induced inflammasome activation in the microvasculature, but not necessarily cardiomyocytes	[171]
Ischemia: 30 min Reperfusion: 24 and 48 h	NLRP3 Inflammasome	Mice Administration of 16673-34-0 (NLRP3 inflammasome inhibitor)	↓ infarct size ↓ cardiac injury	Inhibition of NLRP3 inflammasome is cardioprotective	[172]
Ischemia: 30 min Reperfusion: 3 and 24 h	Gasdermin D (GSDMD), pyroptosis	Mice GSDMD^-/-^ global KO	↓ infarct size ↓ cardiac injury ↓ cardiomyocyte death	I/R-induced oxidative stress activates and cleaves GSDMD and kills cardiomyocytes through pyroptosis	[173]
Ischemia: 30 min Reperfusion: 1, 3, 6, and 12 h 1, 3, and 7 days	S100a9 alarmins	Mice S100a9 transgenic S100a9 global KO Administration of S100a9 neutralizing antibodies	S100a9 TG: ↑ infarct size ↑ fibrosis ↓ cardiac function S100a9 KO or Abs: ↓ infarct size ↓ fibrosis ↑ cardiac function	Altered ETC complex I expression and activity in cardiomyocytes modulates I/R injury	[174]
Ischemia: 45 min Reperfusion: 3 h, 45 days	Neutrophil extracellular traps (NETs)	Rats Administration of DNase I +/- plasminogen activator	↓ infarct size ↓ pathological remodeling ↓ no reflow ↑ cardiac function	Cleavage/reduction in NETs with DNAse I treatment, decreased MPO activity, and reduced thrombosis afforded cardioprotection	[175]
Ischemia: 45 min Reperfusion: 4 h, 3, 7, 14, and 28 days	Macrophage, MerTK	Mice MerTK myeloid KO	↑ infarct size ↓ cardiac function	Cleavage of MerTK on resident macrophages during I/R enhances injury and suppresses repair	[103]
Ischemia: 45 min Reperfusion: 24 h	Dectin-1	Mice Dectin 1^-/-^ global KO	↓ infarct size ↓ immune cell infiltration ↑ cardiac function	Dectin-1 positively regulates NF-κB signaling, inflammatory cytokines, and neutrophil recruitment	[176]
Ischemia: 40 min Reperfusion: 60 min	Plasmacytoid dendritic cells (pDC)	Mice Administration of pDC antigen-1 antibody depletion	↓ infarct size ↓ cardiac injury	Suppressed secretion of Type 1 interferons	[120]
Ischemia: 30 min Reperfusion: 180 min	Dendritic cells, HMGB1	Rats Administration of HMGB1 neutralizing antibodies	↓ infarct size ↓ cardiac injury ↑ cardiac function	Suppression of inflammatory dendritic cell recruitment and cardiomyocyte apoptosis	[123]
Ischemia: 30 min Reperfusion: 3, 7, and 14 days	Tregs, IL-2 signaling	Mice Use of PC61 (CD25) neutralizing antibodies	↑ infarct size ↑ cardiac injury ↑ cardiac remodeling ↓ cardiac function	IL-2C Treg suppression enhances inflammation and injury	[177]
Ischemia: 45 min Reperfusion: 15 min, 60 min, 24 h	T cells CD4+ and CD8+ subtypes	Mice Administration of CD4 or CD8 neutralizing antibodies	CD4 depletion: ↓ infarct size ↓ leukocyte recruitment CD8 depletion: No change in infarct	CD4 T cells regulate IFNγ, and inflammatory cell recruitment	[135]
Ischemia: 90 min, closed chest Reperfusion: 2 weeks	B cell/ Total depletion	Mice Administration of CD20 neutralizing antibodies or Pirfenidone	Survival advantage after I/R, but no significant difference in cardiac function	Pirfenidone-mediated reduction in B cell infiltration and inflammation may afford benefit	[152]
Ischemia: 1 h Reperfusion: 24 h	B cell/ IgM activation	Mice Cr2^-/-^ global KO RAG1^-/-^ global KO	↓ infarct size ↓ neutrophil recruitment ↑ cardiac function	I/R-induced autoactivation of B cells is mediated by IgM and contributes to I/R injury	[178]

## Data Availability

No new data were created or analyzed in this study. Data sharing is not applicable to this article.

## References

[1] Elgendy I.Y., Mahtta D., Pepine C.J. (2019). Medical Therapy for Heart Failure Caused by Ischemic Heart Disease. Circ. Res..

[2] Virani S.S., Alonso A., Benjamin E.J., Bittencourt M.S., Callaway C.W., Carson A.P., Chamberlain A.M., Chang A.R., Cheng S., Delling F.N. (2020). Heart Disease and Stroke Statistics—2020 Update: A Report from the American Heart Association. Circulation.

[3] Liu Y., Li L., Wang Z., Zhang J., Zhou Z. (2023). Myocardial ischemia-reperfusion injury; Molecular mechanisms and prevention. Microvasc. Res..

[4] Zhang R.Y.K., Cochran B.J., Thomas S.R., Rye K.A. (2023). Impact of Reperfusion on Temporal Immune Cell Dynamics after Myocardial Infarction. J. Am. Heart Assoc..

[5] Silvis M.J.M., Dengler S.E.K.G., Odille C.A., Mishra M., van der Kaaij N.P., Doevendans P.A., Sluijter J.P.G., de Kleijn D.P.V., de Jager S.C.A., Bosch L. (2020). Damage-Associated Molecular Patterns in Myocardial Infarction and Heart Transplantation: The Road to Translational Success. Front. Immunol..

[6] Giordano F.J. (2005). Oxygen; oxidative stress; hypoxia; heart failure. J. Clin. Investig..

[7] Hausenloy D.J., Botker H.E., Engstrom T., Erlinge D., Heusch G., Ibanez B., Kloner R.A., Ovize M., Yellon D.M., Garcia-Dorado D. (2017). Targeting reperfusion injury in patients with ST-segment elevation myocardial infarction: Trials and tribulations. Eur. Heart J..

[8] Hausenloy D.J., Yellon D.M. (2013). Myocardial ischemia-reperfusion injury: A neglected therapeutic target. J. Clin. Investig..

[9] Ibáñez B., Heusch G., Ovize M., Van de Werf F. (2015). Evolving Therapies for Myocardial Ischemia/Reperfusion Injury. J. Am. Coll. Cardiol..

[10] Schirone L., Forte M., D’Ambrosio L., Valenti V., Vecchio D., Schiavon S., Spinosa G., Sarto G., Petrozza V., Frati G. (2022). An Overview of the Molecular Mechanisms Associated with Myocardial Ischemic Injury: State of the Art and Translational Perspectives. Cells.

[11] Del Re D.P., Amgalan D., Linkermann A., Liu Q., Kitsis R.N. (2019). Fundamental Mechanisms of Regulated Cell Death and Implications for Heart Disease. Physiol. Rev..

[12] Hashimoto H., Olson E.N., Bassel-Duby R. (2018). Therapeutic approaches for cardiac regeneration and repair. Nat. Rev. Cardiol..

[13] Swirski F.K., Nahrendorf M. (2018). Cardioimmunology: The immune system in cardiac homeostasis and disease. Nat. Rev. Immunol..

[14] Vagnozzi R.J., Maillet M., Sargent M.A., Khalil H., Johansen A.K.Z., Schwanekamp J.A., York A.J., Huang V., Nahrendorf M., Sadayappan S. (2020). An acute immune response underlies the benefit of cardiac stem cell therapy. Nature.

[15] Zuurbier C.J., Abbate A., Cabrera-Fuentes H.A., Cohen M.V., Collino M., De Kleijn D.P.V., Downey J.M., Pagliaro P., Preissner K.T., Takahashi M. (2019). Innate immunity as a target for acute cardioprotection. Cardiovasc. Res..

[16] Andreadou I., Cabrera-Fuentes H.A., Devaux Y., Frangogiannis N.G., Frantz S., Guzik T., Liehn E.A., Gomes C.P.C., Schulz R., Hausenloy D.J. (2019). Immune cells as targets for cardioprotection: New players and novel therapeutic opportunities. Cardiovasc. Res..

[17] Gordon J.W., Shaw J.A., Kirshenbaum L.A. (2011). Multiple Facets of NF-kB in the Heart. Circ. Res..

[18] Oyama J., Blais C., Liu X., Pu M., Kobzik L., Kelly R.A., Bourcier T. (2004). Reduced myocardial ischemia-reperfusion injury in toll-like receptor 4-deficient mice. Circulation.

[19] Toldo S., Mauro A.G., Cutter Z., Abbate A. (2018). Inflammasome; pyroptosis, and cytokines in myocardial ischemia-reperfusion injury. Am. J. Physiol.-Heart Circ. Physiol..

[20] Turner N.A. (2016). Inflammatory and fibrotic responses of cardiac fibroblasts to myocardial damage associated molecular patterns (DAMPs). J. Mol. Cell. Cardiol..

[21] Fujiwara M., Matoba T., Koga J.-I., Okahara A., Funamoto D., Nakano K., Tsutsui H., Egashira K. (2019). Nanoparticle incorporating Toll-like receptor 4 inhibitor attenuates myocardial ischaemia–reperfusion injury by inhibiting monocyte-mediated inflammation in mice. Cardiovasc. Res..

[22] Timmers L., Sluijter J.P., van Keulen J.K., Hoefer I.E., Nederhoff M.G., Goumans M.J., Doevendans P.A., van Echteld C.J., Joles J.A., Quax P.H. (2008). Toll-like receptor 4 mediates maladaptive left ventricular remodeling and impairs cardiac function after myocardial infarction. Circ. Res..

[23] Arslan F., Smeets M.B., O’Neill L.A.J., Keogh B., McGuirk P., Timmers L., Tersteeg C., Hoefer I.E., Doevendans P.A., Pasterkamp G. (2010). Myocardial Ischemia/Reperfusion Injury Is Mediated by Leukocytic Toll-Like Receptor-2 and Reduced by Systemic Administration of a Novel Anti–Toll-Like Receptor-2 Antibody. Circulation.

[24] Shao Y., Saredy J., Yang W.Y., Sun Y., Lu Y., Saaoud F., Drummer C., Johnson C., Xu K., Jiang X. (2020). Vascular Endothelial Cells and Innate Immunity. Arterioscler. Thromb. Vasc. Biol..

[25] Bucciarelli L.G., Kaneko M., Ananthakrishnan R., Harja E., Lee L.K., Hwang Y.C., Lerner S., Bakr S., Li Q., Lu Y. (2006). Receptor for advanced-glycation end products: Key modulator of myocardial ischemic injury. Circulation.

[26] Hill J.H., Ward P.A. (1971). The phlogistic role of C3 leukotactic fragments in myocardial infarcts of rats. J. Exp. Med..

[27] Cao D.J., Schiattarella G.G., Villalobos E., Jiang N., May H.I., Li T., Chen Z.J., Gillette T.G., Hill J.A. (2018). Cytosolic DNA Sensing Promotes Macrophage Transformation and Governs Myocardial Ischemic Injury. Circulation.

[28] Lu C., Ren D., Wang X., Ha T., Liu L., Lee E.J., Hu J., Kalbfleisch J., Gao X., Kao R. (2014). Toll-like receptor 3 plays a role in myocardial infarction and ischemia/reperfusion injury. Biochim. Biophys. Acta.

[29] Del Re D.P. (2022). Hippo-Yap signaling in cardiac and fibrotic remodeling. Curr. Opin. Physiol..

[30] Schulz R. (2008). TNFα in myocardial ischemia/reperfusion: Damage vs. protection. J. Mol. Cell. Cardiol..

[31] Margraf A., Ley K., Zarbock A. (2019). Neutrophil Recruitment: From Model Systems to Tissue-Specific Patterns. Trends Immunol..

[32] Hara A., Tallquist M.D. (2023). Fibroblast and Immune Cell Cross-Talk in Cardiac Fibrosis. Curr. Cardiol. Rep..

[33] Thomas T.P., Grisanti L.A. (2020). The Dynamic Interplay Between Cardiac Inflammation and Fibrosis. Front. Physiol..

[34] Francisco J., Zhang Y., Nakada Y., Jeong J.I., Huang C.Y., Ivessa A., Oka S., Babu G.J., Del Re D.P. (2021). AAV-mediated YAP expression in cardiac fibroblasts promotes inflammation and increases fibrosis. Sci. Rep..

[35] Sreejit G., Nooti S.K., Jaggers R.M., Athmanathan B., Park K.H., Al-Sharea A., Johnson J., Dahdah A., Lee M.K.S., Ma J. (2022). Retention of the NLRP3 Inflammasome-Primed Neutrophils in the Bone Marrow Is Essential for Myocardial Infarction-Induced Granulopoiesis. Circulation.

[36] Vogt K.L., Summers C., Chilvers E.R., Condliffe A.M. (2018). Priming and de-priming of neutrophil responses in vitro and in vivo. Eur. J. Clin. Investig..

[37] Miralda I., Uriarte S.M., McLeish K.R. (2017). Multiple Phenotypic Changes Define Neutrophil Priming. Front. Cell Infect. Microbiol..

[38] Schloss M.J., Horckmans M., Nitz K., Duchene J., Drechsler M., Bidzhekov K., Scheiermann C., Weber C., Soehnlein O., Steffens S. (2016). The time-of-day of myocardial infarction onset affects healing through oscillations in cardiac neutrophil recruitment. EMBO Mol. Med..

[39] Puhl S.L., Steffens S. (2019). Neutrophils in Post-myocardial Infarction Inflammation: Damage vs. Resolution?. Front. Cardiovasc. Med..

[40] Hoyer F.F., Nahrendorf M. (2017). Neutrophil contributions to ischaemic heart disease. Eur. Heart J..

[41] Ma Y., Yabluchanskiy A., Iyer R.P., Cannon P.L., Flynn E.R., Jung M., Henry J., Cates C.A., Deleon-Pennell K.Y., Lindsey M.L. (2016). Temporal neutrophil polarization following myocardial infarction. Cardiovasc. Res..

[42] Ma Y., Yabluchanskiy A., Lindsey M.L. (2013). Neutrophil roles in left ventricular remodeling following myocardial infarction. Fibrogenesis Tissue Repair.

[43] Silvestre-Roig C., Braster Q., Ortega-Gomez A., Soehnlein O.A.-O. (2020). Neutrophils as regulators of cardiovascular inflammation. Nat. Rev. Cardiol..

[44] Zhou Z., Zhang S., Ding S., Abudupataer M., Zhang Z., Zhu X., Zhang W., Zou Y., Yang X.A.-O., Ge J.A.-O. (2019). Excessive Neutrophil Extracellular Trap Formation Aggravates Acute Myocardial Infarction Injury in Apolipoprotein E Deficiency Mice via the ROS-Dependent Pathway. Oxid. Med. Cell. Longev..

[45] Dang P.M., Stensballe A., Boussetta T., Raad H., Dewas C., Kroviarski Y., Hayem G., Jensen O.N., Gougerot-Pocidalo M.A., El-Benna J. (2006). A specific p47phox-serine phosphorylated by convergent MAPKs mediates neutrophil NADPH oxidase priming at inflammatory sites. J. Clin. Investig..

[46] Calcagno D.M., Zhang C., Toomu A., Huang K., Ninh V.K., Miyamoto S., Aguirre A.D., Fu Z., Brown J.H., King K.R. (2021). SiglecF(HI) Marks Late-Stage Neutrophils of the Infarcted Heart: A Single-Cell Transcriptomic Analysis of Neutrophil Diversification. J. Am. Heart Assoc..

[47] Kitchen E., Rossi A.G., Condliffe A.M., Haslett C., Chilvers E.R. (1996). Demonstration of reversible priming of human neutrophils using platelet-activating factor. Blood.

[48] Vafadarnejad E., Rizzo G., Krampert L., Arampatzi P., Arias-Loza A.-P., Nazzal Y., Rizakou A., Knochenhauer T., Bandi S.R., Nugroho V.A. (2020). Dynamics of Cardiac Neutrophil Diversity in Murine Myocardial Infarction. Circ. Res..

[49] Sano S., Wang Y., Yura Y., Sano M., Oshima K., Yang Y., Katanasaka Y., Min K.D., Matsuura S., Ravid K. (2019). *JAK2^V617F^*—Mediated Clonal Hematopoiesis Accelerates Pathological Remodeling in Murine Heart Failure. JACC Basic. Transl. Sci..

[50] Kain V., Halade G.V. (2020). Role of neutrophils in ischemic heart failure. Pharmacol. Ther..

[51] Hurtado-Nedelec M., Csillag-Grange M.J., Boussetta T., Belambri S.A., Fay M., Cassinat B., Gougerot-Pocidalo M.A., Dang P.M., El-Benna J. (2013). Increased reactive oxygen species production and p47phox phosphorylation in neutrophils from myeloproliferative disorders patients with JAK2 (V617F) mutation. Haematologica.

[52] Vinten-Johansen J. (2004). Involvement of neutrophils in the pathogenesis of lethal myocardial reperfusion injury. Cardiovasc. Res..

[53] Horckmans M., Ring L., Duchene J., Santovito D., Schloss M.J., Drechsler M., Weber C., Soehnlein O., Steffens S. (2017). Neutrophils orchestrate post-myocardial infarction healing by polarizing macrophages towards a reparative phenotype. Eur. Heart J..

[54] Winterbourn C.C., Kettle A.J., Hampton M.B. (2016). Reactive Oxygen Species and Neutrophil Function. Annu. Rev. Biochem..

[55] Gierlikowska B., Stachura A., Gierlikowski W., Demkow U. (2021). Phagocytosis, Degranulation and Extracellular Traps Release by Neutrophils-The Current Knowledge, Pharmacological Modulation and Future Prospects. Front. Pharmacol..

[56] Naegelen I., Beaume N., Plançon S., Schenten V., Tschirhart E.J., Bréchard S. (2015). Regulation of Neutrophil Degranulation and Cytokine Secretion: A Novel Model Approach Based on Linear Fitting. J. Immunol. Res..

[57] García-Prieto J., Villena-Gutiérrez R., Gómez M., Bernardo E., Pun-García A., García-Lunar I., Crainiciuc G., Fernández-Jiménez R., Sreeramkumar V., Bourio-Martínez R. (2017). Neutrophil stunning by metoprolol reduces infarct size. Nat. Commun..

[58] Entman M.L., Youker K., Shoji T., Kukielka G., Shappell S.B., Taylor A.A., Smith C.W. (1992). Neutrophil induced oxidative injury of cardiac myocytes. A compartmented system requiring CD11b/CD18-ICAM-1 adherence. J. Clin. Investig..

[59] Kloner R.A., Ganote C.E., Jennings R.B. (1974). The “no-reflow” phenomenon after temporary coronary occlusion in the dog. J. Clin. Investig..

[60] Halade G.V., Lee D.H. (2022). Inflammation and resolution signaling in cardiac repair and heart failure. EBioMedicine.

[61] Dehghani T., Thai P.N., Sodhi H., Ren L., Sirish P., Nader C.E., Timofeyev V., Overton J.L., Li X., Lam K.S. (2020). Selectin-Targeting Glycosaminoglycan-Peptide Conjugate Limits Neutrophil Mediated Cardiac Reperfusion Injury. Cardiovasc. Res..

[62] Du M., Yang W., Schmull S., Gu J., Xue S. (2020). Inhibition of peptidyl arginine deiminase-4 protects against myocardial infarction induced cardiac dysfunction. Int. Immunopharmacol..

[63] Ogura Y., Tajiri K.A.-O., Murakoshi N., Xu D., Yonebayashi S., Li S., Okabe Y., Feng D., Shimoda Y., Song Z. (2021). Neutrophil Elastase Deficiency Ameliorates Myocardial Injury Post Myocardial Infarction in Mice. Int. J. Mol. Sci..

[64] Trevelin S.C., Shah A.M. (2020). Lombardi, GBeyond bacterial killing: NADPH oxidase 2 is an immunomodulator. Immunol. Lett..

[65] Ao L., Zou N., Cleveland J.C., Fullerton D.A., Meng X. (2009). Myocardial TLR4 is a determinant of neutrophil infiltration after global myocardial ischemia: Mediating KC and MCP-1 expression induced by extracellular HSC70. Am. J. Physiol.-Heart Circ. Physiol..

[66] Mauler M., Herr N., Schoenichen C., Witsch T., Marchini T., Härdtner C., Koentges C., Kienle K., Ollivier V., Schell M. (2019). Platelet Serotonin Aggravates Myocardial Ischemia/Reperfusion Injury via Neutrophil Degranulation. Circulation.

[67] Daseke M.J., Chalise U., Becirovic-Agic M., Salomon J.D., Cook L.M., Case A.J., Lindsey M.L. (2021). Neutrophil signaling during myocardial infarction wound repair. Cell. Signal..

[68] Ma Y. (2021). Role of Neutrophils in Cardiac Injury and Repair Following Myocardial Infarction. Cells.

[69] Serhan C.N. (2014). Pro-resolving lipid mediators are leads for resolution physiology. Nature.

[70] Kain V., Ingle K.A., Kabarowski J., Barnes S., Limdi N.A., Prabhu S.D., Halade G.V. (2018). Genetic deletion of 12/15 lipoxygenase promotes effective resolution of inflammation following myocardial infarction. J. Mol. Cell Cardiol..

[71] Halade G.V., Kain V., Ingle K.A., Prabhu S.D. (2017). Interaction of 12/15-lipoxygenase with fatty acids alters the leukocyte kinetics leading to improved postmyocardial infarction healing. Am. J. Physiol.-Heart Circ. Physiol..

[72] Wei X., Zou S., Xie Z., Wang Z., Huang N., Cen Z., Hao Y., Zhang C., Chen Z., Zhao F. (2022). EDIL3 deficiency ameliorates adverse cardiac remodelling by neutrophil extracellular traps (NET)-mediated macrophage polarization. Cardiovasc. Res..

[73] Zhang S., Weinberg S., DeBerge M., Gainullina A., Schipma M., Kinchen J.M., Ben-Sahra I., Gius D.R., Yvan-Charvet L., Chandel N.S. (2019). Efferocytosis Fuels Requirements of Fatty Acid Oxidation and the Electron Transport Chain to Polarize Macrophages for Tissue Repair. Cell Metab..

[74] Chambers S.E., O’Neill C.L., O’Doherty T.M., Medina R.J., Stitt A.W. (2013). The role of immune-related myeloid cells in angiogenesis. Immunobiology.

[75] Davidson S.M., Ferdinandy P., Andreadou I., Bøtker H.E., Heusch G., Ibáñez B., Ovize M., Schulz R., Yellon D.M., Hausenloy D.J. (2019). Multitarget Strategies to Reduce Myocardial Ischemia/Reperfusion Injury: JACC Review Topic of the Week. J. Am. Coll. Cardiol..

[76] Piccolo E.B., Thorp E.B., Sumagin R. (2022). Functional implications of neutrophil metabolism during ischemic tissue repair. Curr. Opin. Pharmacol..

[77] Rodriguez-Espinosa O., Rojas-Espinosa O., Moreno-Altamirano M.M., Lopez-Villegas E.O., Sanchez-Garcia F.J. (2015). Metabolic requirements for neutrophil extracellular traps formation. Immunology.

[78] Britt E.C., Lika J., Giese M.A., Schoen T.J., Seim G.L., Huang Z., Lee P.Y., Huttenlocher A., Fan J. (2022). Switching to the cyclic pentose phosphate pathway powers the oxidative burst in activated neutrophils. Nat. Metab..

[79] Bao Y., Ledderose C., Graf A.F., Brix B., Birsak T., Lee A., Zhang J., Junger W.G. (2015). mTOR and differential activation of mitochondria orchestrate neutrophil chemotaxis. J. Cell Biol..

[80] Bao Y., Ledderose C., Seier T., Graf A.F., Brix B., Chong E., Junger W.G. (2014). Mitochondria regulate neutrophil activation by generating ATP for autocrine purinergic signaling. J. Biol. Chem..

[81] Chen Y., Corriden R., Inoue Y., Yip L., Hashiguchi N., Zinkernagel A., Nizet V., Insel P.A., Junger W.G. (2006). ATP release guides neutrophil chemotaxis via P2Y2 and A3 receptors. Science.

[82] Zaman R., Hamidzada H., Epelman S. (2021). Exploring cardiac macrophage heterogeneity in the healthy and diseased myocardium. Curr. Opin. Immunol..

[83] Pinto A.R., Ilinykh A., Ivey M.J., Kuwabara J.T., D’Antoni M.L., Debuque R., Chandran A., Wang L., Arora K., Rosenthal N.A. (2016). Revisiting Cardiac Cellular Composition. Circ. Res..

[84] Lavine K.J., Pinto A.R., Epelman S., Kopecky B.J., Clemente-Casares X., Godwin J., Rosenthal N., Kovacic J.C. (2018). The Macrophage in Cardiac Homeostasis and Disease: JACC Macrophage in CVD Series (Part 4). J. Am. Coll. Cardiol..

[85] Zaman R., Epelman S. (2022). Resident cardiac macrophages: Heterogeneity and function in health and disease. Immunity.

[86] Kubota A., Frangogiannis N.G. (2022). Macrophages in myocardial infarction. Am. J. Physiol. Cell Physiol..

[87] Ma Y., Mouton A.J., Lindsey M.L. (2018). Cardiac macrophage biology in the steady-state heart, the aging heart, and following myocardial infarction. Transl. Res..

[88] Bajpai G., Schneider C., Wong N., Bredemeyer A., Hulsmans M., Nahrendorf M., Epelman S., Kreisel D., Liu Y., Itoh A. (2018). The human heart contains distinct macrophage subsets with divergent origins and functions. Nat. Med..

[89] Francisco J., Guan J., Zhang Y., Nakada Y., Mareedu S., Sung E.A., Hu C.M., Oka S., Zhai P., Sadoshima J. (2023). Suppression of myeloid YAP antagonizes adverse cardiac remodeling during pressure overload stress. J. Mol. Cell Cardiol..

[90] Dick S.A., Macklin J.A., Nejat S., Momen A., Clemente-Casares X., Althagafi M.G., Chen J., Kantores C., Hosseinzadeh S., Aronoff L. (2019). Self-renewing resident cardiac macrophages limit adverse remodeling following myocardial infarction. Nat. Immunol..

[91] Chakarov S., Lim H.Y., Tan L., Lim S.Y., See P., Lum J., Zhang X.M., Foo S., Nakamizo S., Duan K. (2019). Two distinct interstitial macrophage populations coexist across tissues in specific subtissular niches. Science.

[92] Nicolás-Ávila J.A., Lechuga-Vieco A.V., Esteban-Martínez L., Sánchez-Díaz M., Díaz-García E., Santiago D.J., Rubio-Ponce A., Li J.L., Balachander A., Quintana J.A. (2020). A Network of Macrophages Supports Mitochondrial Homeostasis in the Heart. Cell.

[93] Leid J., Carrelha J., Boukarabila H., Epelman S., Jacobsen S.E., Lavine K.J. (2016). Primitive Embryonic Macrophages are Required for Coronary Development and Maturation. Circ. Res..

[94] Leuschner F., Rauch P.J., Ueno T., Gorbatov R., Marinelli B., Lee W.W., Dutta P., Wei Y., Robbins C., Iwamoto Y. (2012). Rapid monocyte kinetics in acute myocardial infarction are sustained by extramedullary monocytopoiesis. J. Exp. Med..

[95] Bajpai G., Bredemeyer A., Li W., Zaitsev K., Koenig A.L., Lokshina I., Mohan J., Ivey B., Hsiao H.-M., Weinheimer C. (2019). Tissue Resident CCR2− and CCR2+ Cardiac Macrophages Differentially Orchestrate Monocyte Recruitment and Fate Specification Following Myocardial Injury. Circ. Res..

[96] Yan X., Anzai A., Katsumata Y., Matsuhashi T., Ito K., Endo J., Yamamoto T., Takeshima A., Shinmura K., Shen W. (2013). Temporal dynamics of cardiac immune cell accumulation following acute myocardial infarction. J. Mol. Cell. Cardiol..

[97] Majmudar M.D., Keliher E.J., Heidt T., Leuschner F., Truelove J., Sena B.F., Gorbatov R., Iwamoto Y., Dutta P., Wojtkiewicz G. (2013). Monocyte-directed RNAi targeting CCR2 improves infarct healing in atherosclerosis-prone mice. Circulation.

[98] Lantz C., Radmanesh B., Liu E., Thorp E.B., Lin J. (2020). Single-cell RNA sequencing uncovers heterogenous transcriptional signatures in macrophages during efferocytosis. Sci. Rep..

[99] Heidt T., Courties G., Dutta P., Sager H.B., Sebas M., Iwamoto Y., Sun Y., Da Silva N., Panizzi P., van der Laan A.M. (2014). Differential contribution of monocytes to heart macrophages in steady-state and after myocardial infarction. Circ. Res..

[100] DeBerge M., Chaudhary R., Schroth S., Thorp E.B. (2023). Immunometabolism at the Heart of Cardiovascular Disease. JACC Basic. Transl. Sci..

[101] Zhang S., Bories G., Lantz C., Emmons R., Becker A., Liu E., Abecassis M.M., Yvan-Charvet L., Thorp E.B. (2019). Immunometabolism of Phagocytes and Relationships to Cardiac Repair. Front. Cardiovasc. Med..

[102] Mouton A.J., DeLeon-Pennell K.Y., Gonzalez O.J.R., Flynn E.R., Freeman T.C., Saucerman J.J., Garrett M.R., Ma Y., Harmancey R., Lindsey M.L. (2018). Mapping macrophage polarization over the myocardial infarction time continuum. Basic. Res. Cardiol..

[103] DeBerge M., Yeap X.Y., Dehn S., Zhang S., Grigoryeva L., Misener S., Procissi D., Zhou X., Lee D.C., Muller W.A. (2017). MerTK Cleavage on Resident Cardiac Macrophages Compromises Repair After Myocardial Ischemia Reperfusion Injury. Circ. Res..

[104] Tannahill G.M., Curtis A.M., Adamik J., Palsson-McDermott E.M., McGettrick A.F., Goel G., Frezza C., Bernard N.J., Kelly B., Foley N.H. (2013). Succinate is an inflammatory signal that induces IL-1beta through HIF-1alpha. Nature.

[105] Wan E., Yeap X.Y., Dehn S., Terry R., Novak M., Zhang S., Iwata S., Han X., Homma S., Drosatos K. (2013). Enhanced efferocytosis of apoptotic cardiomyocytes through myeloid-epithelial-reproductive tyrosine kinase links acute inflammation resolution to cardiac repair after infarction. Circ. Res..

[106] Chouchani E.T., Pell V.R., Gaude E., Aksentijevic D., Sundier S.Y., Robb E.L., Logan A., Nadtochiy S.M., Ord E.N., Smith A.C. (2014). Ischaemic accumulation of succinate controls reperfusion injury through mitochondrial ROS. Nature.

[107] Mills E.L., Kelly B., Logan A., Costa A.S.H., Varma M., Bryant C.E., Tourlomousis P., Dabritz J.H.M., Gottlieb E., Latorre I. (2016). Succinate Dehydrogenase Supports Metabolic Repurposing of Mitochondria to Drive Inflammatory Macrophages. Cell.

[108] Wang F., Zhang S., Vuckovic I., Jeon R., Lerman A., Folmes C.D., Dzeja P.P., Herrmann J. (2018). Glycolytic Stimulation Is Not a Requirement for M2 Macrophage Differentiation. Cell Metab..

[109] Mills E.L., Ryan D.G., Prag H.A., Dikovskaya D., Menon D., Zaslona Z., Jedrychowski M.P., Costa A.S.H., Higgins M., Hams E. (2018). Itaconate is an anti-inflammatory metabolite that activates Nrf2 via alkylation of KEAP1. Nature.

[110] Hooftman A., Angiari S., Hester S., Corcoran S.E., Runtsch M.C., Ling C., Ruzek M.C., Slivka P.F., McGettrick A.F., Banahan K. (2020). The Immunomodulatory Metabolite Itaconate Modifies NLRP3 and Inhibits Inflammasome Activation. Cell Metab..

[111] Lampropoulou V., Sergushichev A., Bambouskova M., Nair S., Vincent E.E., Loginicheva E., Cervantes-Barragan L., Ma X., Huang S.C., Griss T. (2016). Itaconate Links Inhibition of Succinate Dehydrogenase with Macrophage Metabolic Remodeling and Regulation of Inflammation. Cell Metab..

[112] Hsu C.P., Oka S., Shao D., Hariharan N., Sadoshima J. (2009). Nicotinamide phosphoribosyltransferase regulates cell survival through NAD+ synthesis in cardiac myocytes. Circ. Res..

[113] Yamamoto T., Byun J., Zhai P., Ikeda Y., Oka S., Sadoshima J. (2014). Nicotinamide mononucleotide, an intermediate of NAD+ synthesis, protects the heart from ischemia and reperfusion. PLoS ONE.

[114] Zhai X., Han W., Wang M., Guan S., Qu X. (2019). Exogenous supplemental NAD+ protect myocardium against myocardial ischemic/reperfusion injury in swine model. Am. J. Transl. Res..

[115] Zhou B., Wang D.D., Qiu Y., Airhart S., Liu Y., Stempien-Otero A., O’Brien K.D., Tian R. (2020). Boosting NAD level suppresses inflammatory activation of PBMCs in heart failure. J. Clin. Investig..

[116] Lee C.F., Caudal A., Abell L., Gowda G.A.N., Tian R. (2019). Targeting NAD(+) Metabolism as Interventions for Mitochondrial Disease. Sci. Rep..

[117] Vats D., Mukundan L., Odegaard J.I., Zhang L., Smith K.L., Morel C.R., Wagner R.A., Greaves D.R., Murray P.J., Chawla A. (2006). Oxidative metabolism and PGC-1beta attenuate macrophage-mediated inflammation. Cell Metab..

[118] Cai S., Zhao M., Zhou B., Yoshii A., Bugg D., Villet O., Sahu A., Olson G.S., Davis J., Tian R. (2023). Mitochondrial dysfunction in macrophages promotes inflammation and suppresses repair after myocardial infarction. J. Clin. Investig..

[119] Morioka S., Perry J.S.A., Raymond M.H., Medina C.B., Zhu Y., Zhao L., Serbulea V., Onengut-Gumuscu S., Leitinger N., Kucenas S. (2018). Efferocytosis induces a novel SLC program to promote glucose uptake and lactate release. Nature.

[120] Lai L., Zhang A., Yang B., Charles E.J., Kron I.L., Yang Z. (2021). Plasmacytoid Dendritic Cells Mediate Myocardial Ischemia/Reperfusion Injury by Secreting Type I Interferons. J. Am. Heart Assoc..

[121] Lee J.S., Jeong S.J., Kim S., Chalifour L., Yun T.J., Miah M.A., Li B., Majdoubi A., Sabourin A., Keler T. (2018). Conventional Dendritic Cells Impair Recovery after Myocardial Infarction. J. Immunol..

[122] Anzai A., Anzai T., Nagai S., Maekawa Y., Naito K., Kaneko H., Sugano Y., Takahashi T., Abe H., Mochizuki S. (2012). Regulatory role of dendritic cells in postinfarction healing and left ventricular remodeling. Circulation.

[123] Xue J., Ge H., Lin Z., Wang H., Lin W., Liu Y., Wu G., Xia J., Zhao Q. (2019). The role of dendritic cells regulated by HMGB1/TLR4 signalling pathway in myocardial ischaemia reperfusion injury. J. Cell Mol. Med..

[124] Choo E.H., Lee J.-H., Park E.-H., Park H.E., Jung N.-C., Kim T.-H., Koh Y.-S., Kim E., Seung K.-B., Park C. (2017). Infarcted Myocardium-Primed Dendritic Cells Improve Remodeling and Cardiac Function After Myocardial Infarction by Modulating the Regulatory T Cell and Macrophage Polarization. Circulation.

[125] Van der Borght K., Scott C.L., Nindl V., Bouché A., Martens L., Sichien D., Van Moorleghem J., Vanheerswynghels M., De Prijck S., Saeys Y. (2017). Myocardial Infarction Primes Autoreactive T Cells through Activation of Dendritic Cells. Cell Rep..

[126] Forte E., Perkins B., Sintou A., Kalkat H.S., Papanikolaou A., Jenkins C., Alsubaie M., Chowdhury R.A., Duffy T.M., Skelly D.A. (2021). Cross-Priming Dendritic Cells Exacerbate Immunopathology After Ischemic Tissue Damage in the Heart. Circulation.

[127] Cohen C.D., Rousseau S.T., Bermea K.C., Bhalodia A., Lovell J.P., Zita M.D., Čiháková D., Adamo L. (2023). Myocardial Immune Cells: The Basis of Cardiac Immunology. J. Immunol..

[128] Lv H., Lipes M.A. (2012). Role of impaired central tolerance to α-myosin in inflammatory heart disease. Trends Cardiovasc. Med..

[129] Thwe P.M., Pelgrom L.R., Cooper R., Beauchamp S., Reisz J.A., D’Alessandro A., Everts B., Amiel E. (2017). Cell-Intrinsic Glycogen Metabolism Supports Early Glycolytic Reprogramming Required for Dendritic Cell Immune Responses. Cell Metab..

[130] Krawczyk C.M., Holowka T., Sun J., Blagih J., Amiel E., DeBerardinis R.J., Cross J.R., Jung E., Thompson C.B., Jones R.G. (2010). Toll-like receptor-induced changes in glycolytic metabolism regulate dendritic cell activation. Blood.

[131] Basit F., Mathan T., Sancho D., de Vries I.J.M. (2018). Human Dendritic Cell Subsets Undergo Distinct Metabolic Reprogramming for Immune Response. Front. Immunol..

[132] Adamo L., Rocha-Resende C., Lin C.Y., Evans S., Williams J., Dun H., Li W., Mpoy C., Andhey P.S., Rogers B.E. (2020). Myocardial B cells are a subset of circulating lymphocytes with delayed transit through the heart. JCI Insight.

[133] Frangogiannis N.G., Mendoza L.H., Lindsey M.L., Ballantyne C.M., Michael L.H., Smith C.W., Entman M.L. (2000). IL-10 is induced in the reperfused myocardium and may modulate the reaction to injury. J. Immunol..

[134] Boag S.E., Das R., Shmeleva E.V., Bagnall A., Egred M., Howard N., Bennaceur K., Zaman A., Keavney B., Spyridopoulos I. (2015). T lymphocytes and fractalkine contribute to myocardial ischemia/reperfusion injury in patients. J. Clin. Investig..

[135] Yang Z., Day Y.J., Toufektsian M.C., Xu Y., Ramos S.I., Marshall M.A., French B.A., Linden J. (2006). Myocardial infarct-sparing effect of adenosine A2A receptor activation is due to its action on CD4+ T lymphocytes. Circulation.

[136] Santos-Zas I., Lemarié J., Zlatanova I., Cachanado M., Seghezzi J.C., Benamer H., Goube P., Vandestienne M., Cohen R., Ezzo M. (2021). Cytotoxic CD8(+) T cells promote granzyme B-dependent adverse post-ischemic cardiac remodeling. Nat. Commun..

[137] Kino T., Khan M., Mohsin S. (2020). The Regulatory Role of T Cell Responses in Cardiac Remodeling Following Myocardial Infarction. Int. J. Mol. Sci..

[138] Bansal S.S., Ismahil M.A., Goel M., Zhou G., Rokosh G., Hamid T., Prabhu S.D. (2019). Dysfunctional and proinflammatory regulatory T-lymphocytes are essential for adverse cardiac remodeling in ischemic cardiomyopathy. Circulation.

[139] Bansal S.S., Ismahil M.A., Goel M., Patel B., Hamid T., Rokosh G., Prabhu S.D. (2017). Activated T lymphocytes are essential drivers of pathological remodeling in ischemic heart failure. Circ. Heart Fail..

[140] Xia N., Lu Y., Gu M., Li N., Liu M., Jiao J., Zhu Z., Li J., Li D., Tang T. (2020). A Unique Population of Regulatory T Cells in Heart Potentiates Cardiac Protection From Myocardial Infarction. Circulation.

[141] Hofmann U., Frantz S. (2015). Role of Lymphocytes in Myocardial Injury, Healing, and Remodeling After Myocardial Infarction. Circ. Res..

[142] Saxena A., Dobaczewski M., Rai V., Haque Z., Chen W., Li N., Frangogiannis N.G. (2014). Regulatory T cells are recruited in the infarcted mouse myocardium and may modulate fibroblast phenotype and function. Am. J. Physiol.-Heart Circ. Physiol..

[143] Weirather J., Hofmann U.D., Beyersdorf N., Ramos G.C., Vogel B., Frey A., Ertl G., Kerkau T., Frantz S. (2014). Foxp3+ CD4+ T cells improve healing after myocardial infarction by modulating monocyte/macrophage differentiation. Circ. Res..

[144] Rieckmann M., Delgobo M., Gaal C., Buchner L., Steinau P., Reshef D., Gil-Cruz C., Horst E.N.T., Kircher M., Reiter T. (2019). Myocardial infarction triggers cardioprotective antigen-specific T helper cell responses. J. Clin. Investig..

[145] Zacchigna S., Martinelli V., Moimas S., Colliva A., Anzini M., Nordio A., Costa A., Rehman M., Vodret S., Pierro C. (2018). Paracrine effect of regulatory T cells promotes cardiomyocyte proliferation during pregnancy and after myocardial infarction. Nat. Commun..

[146] Matsumoto K., Ogawa M., Suzuki J., Hirata Y., Nagai R., Isobe M. (2011). Regulatory T lymphocytes attenuate myocardial infarction-induced ventricular remodeling in mice. Int. Heart J..

[147] Peng M., Yin N., Chhangawala S., Xu K., Leslie C.S., Li M.O. (2016). Aerobic glycolysis promotes T helper 1 cell differentiation through an epigenetic mechanism. Science.

[148] Cretenet G., Clerc I., Matias M., Loisel S., Craveiro M., Oburoglu L., Kinet S., Mongellaz C., Dardalhon V., Taylor N. (2016). Cell surface Glut1 levels distinguish human CD4 and CD8 T lymphocyte subsets with distinct effector functions. Sci. Rep..

[149] Michalek R.D., Gerriets V.A., Jacobs S.R., Macintyre A.N., MacIver N.J., Mason E.F., Sullivan S.A., Nichols A.G., Rathmell J.C. (2011). Cutting edge: Distinct glycolytic and lipid oxidative metabolic programs are essential for effector and regulatory CD4+ T cell subsets. J. Immunol..

[150] Adamo L., Rocha-Resende C., Mann D.L. (2020). The Emerging Role of B Lymphocytes in Cardiovascular Disease. Annu. Rev. Immunol..

[151] Rocha-Resende C., Pani F., Adamo L. (2021). B cells modulate the expression of MHC-II on cardiac CCR2^−^ macrophages. J. Mol. Cell Cardiol..

[152] Adamo L., Staloch L.J., Rocha-Resende C., Matkovich S.J., Jiang W., Bajpai G., Weinheimer C.J., Kovacs A., Schilling J.D., Barger P.M. (2018). Modulation of subsets of cardiac B lymphocytes improves cardiac function after acute injury. JCI Insight.

[153] Zouggari Y., Ait-Oufella H., Bonnin P., Simon T., Sage A.P., Guerin C., Vilar J., Caligiuri G., Tsiantoulas D., Laurans L. (2013). B lymphocytes trigger monocyte mobilization and impair heart function after acute myocardial infarction. Nat. Med..

[154] Kyaw T., Loveland P., Kanellakis P., Cao A., Kallies A., Huang A.L., Peter K., Toh B.H., Bobik A. (2021). Alarmin-activated B cells accelerate murine atherosclerosis after myocardial infarction via plasma cell-immunoglobulin-dependent mechanisms. Eur. Heart J..

[155] Goodchild T.T., Robinson K.A., Pang W., Tondato F., Cui J., Arrington J., Godwin L., Ungs M., Carlesso N., Weich N. (2009). Bone Marrow-Derived B Cells Preserve Ventricular Function After Acute Myocardial Infarction. JACC Cardiovasc. Interv..

[156] Wu L., Dalal R., Cao C.D., Postoak J.L., Yang G., Zhang Q., Wang Z., Lal H., Van Kaer L. (2019). IL-10-producing B cells are enriched in murine pericardial adipose tissues and ameliorate the outcome of acute myocardial infarction. Proc. Natl. Acad. Sci. USA.

[157] Caro-Maldonado A., Wang R., Nichols A.G., Kuraoka M., Milasta S., Sun L.D., Gavin A.L., Abel E.D., Kelsoe G., Green D.R. (2014). Metabolic reprogramming is required for antibody production that is suppressed in anergic but exaggerated in chronically BAFF-exposed B cells. J. Immunol..

[158] Kim D.S., Woo J.S., Min H.K., Choi J.W., Moon J.H., Park M.J., Kwok S.K., Park S.H., Cho M.L. (2021). Short-chain fatty acid butyrate induces IL-10-producing B cells by regulating circadian-clock-related genes to ameliorate Sjögren’s syndrome. J. Autoimmun..

[159] Weisel F.J., Mullett S.J., Elsner R.A., Menk A.V., Trivedi N., Luo W., Wikenheiser D., Hawse W.F., Chikina M., Smita S. (2020). Germinal center B cells selectively oxidize fatty acids for energy while conducting minimal glycolysis. Nat. Immunol..

[160] Grisanti L.A., Traynham C.J., Repas A.A., Gao E., Koch W.J., Tilley D.G. (2016). beta2-Adrenergic receptor-dependent chemokine receptor 2 expression regulates leukocyte recruitment to the heart following acute injury. Proc. Natl. Acad. Sci. USA.

[161] van Amerongen M.J., Harmsen M.C., van Rooijen N., Petersen A.H., van Luyn M.J. (2007). Macrophage depletion impairs wound healing and increases left ventricular remodeling after myocardial injury in mice. Am. J. Pathol..

[162] Cao X., Li B., Han X., Zhang X., Dang M., Wang H., Du F., Zeng X., Guo C. (2020). Soluble receptor for advanced glycation end-products promotes angiogenesis through activation of STAT3 in myocardial ischemia/reperfusion injury. Apoptosis.

[163] De Hoog V.C., Timmers L., Van Duijvenvoorde A., De Jager S.C., Van Middelaar B.J., Smeets M.B., Woodruff T.M., Doevendans P.A., Pasterkamp G., Hack C.E. (2014). Leucocyte expression of complement C5a receptors exacerbates infarct size after myocardial reperfusion injury. Cardiovasc. Res..

[164] Vakeva A.P., Agah A., Rollins S.A., Matis L.A., Li L., Stahl G.L. (1998). Myocardial infarction and apoptosis after myocardial ischemia and reperfusion: Role of the terminal complement components and inhibition by anti-C5 therapy. Circulation.

[165] Frantz S., Tillmanns J., Kuhlencordt P.J., Schmidt I., Adamek A., Dienesch C., Thum T., Gerondakis S., Ertl G., Bauersachs J. (2007). Tissue-specific effects of the nuclear factor kappaB subunit p50 on myocardial ischemia-reperfusion injury. Am. J. Pathol..

[166] Zhang X.Q., Tang R., Li L., Szucsik A., Javan H., Saegusa N., Spitzer K.W., Selzman C.H. (2013). Cardiomyocyte-specific p65 NF-κB deletion protects the injured heart by preservation of calcium handling. Am. J. Physiol.-Heart Circ. Physiol..

[167] Moss N.C., Stansfield W.E., Willis M.S., Tang R.-H., Selzman C.H. (2007). IKKβ inhibition attenuates myocardial injury and dysfunction following acute ischemia-reperfusion injury. Am. J. Physiol.-Heart Circ. Physiol..

[168] Maekawa N., Wada H., Kanda T., Niwa T., Yamada Y., Saito K., Fujiwara H., Sekikawa K., Seishima M. (2002). Improved myocardial ischemia/reperfusion injury in mice lacking tumor necrosis factor-alpha. J. Am. Coll. Cardiol..

[169] Kawaguchi M., Takahashi M., Hata T., Kashima Y., Usui F., Morimoto H., Izawa A., Takahashi Y., Masumoto J., Koyama J. (2011). Inflammasome activation of cardiac fibroblasts is essential for myocardial ischemia/reperfusion injury. Circulation.

[170] Toldo S., Marchetti C., Mauro A.G., Chojnacki J., Mezzaroma E., Carbone S., Zhang S., Van Tassell B., Salloum F.N., Abbate A. (2016). Inhibition of the NLRP3 inflammasome limits the inflammatory injury following myocardial ischemia-reperfusion in the mouse. Int. J. Cardiol..

[171] Liu Y., Lian K., Zhang L., Wang R., Yi F., Gao C., Xin C., Zhu D., Li Y., Yan W. (2014). TXNIP mediates NLRP3 inflammasome activation in cardiac microvascular endothelial cells as a novel mechanism in myocardial ischemia/reperfusion injury. Basic. Res. Cardiol..

[172] Marchetti C., Chojnacki J., Toldo S., Mezzaroma E., Tranchida N., Rose S.W., Federici M., Van Tassell B.W., Zhang S., Abbate A. (2014). A novel pharmacologic inhibitor of the NLRP3 inflammasome limits myocardial injury after ischemia-reperfusion in the mouse. J. Cardiovasc. Pharmacol..

[173] Shi H., Gao Y., Dong Z., Yang J., Gao R., Li X., Zhang S., Ma L., Sun X., Wang Z. (2021). GSDMD-Mediated Cardiomyocyte Pyroptosis Promotes Myocardial I/R Injury. Circ. Res..

[174] Li Y., Chen B., Yang X., Zhang C., Jiao Y., Li P., Liu Y., Li Z., Qiao B., Lau W.B. (2019). S100a8/a9 Signaling Causes Mitochondrial Dysfunction and Cardiomyocyte Death in Response to Ischemic/Reperfusion Injury. Circulation.

[175] Ge L., Zhou X., Ji W.-J., Lu R.-Y., Zhang Y., Zhang Y.-D., Ma Y.-Q., Zhao J.-H., Li Y.-M. (2015). Neutrophil extracellular traps in ischemia-reperfusion injury-induced myocardial no-reflow: Therapeutic potential of DNase-based reperfusion strategy. Am. J. Physiol.-Heart Circ. Physiol..

[176] Fan Q., Tao R., Zhang H., Xie H., Lu L., Wang T., Su M., Hu J., Zhang Q., Chen Q. (2019). Dectin-1 Contributes to Myocardial Ischemia/Reperfusion Injury by Regulating Macrophage Polarization and Neutrophil Infiltration. Circulation.

[177] Xiao J., Yu K., Li M., Xiong C., Wei Y., Zeng Q. (2017). The IL-2/Anti-IL-2 Complex Attenuates Cardiac Ischaemia-Reperfusion Injury Through Expansion of Regulatory T Cells. Cell Physiol. Biochem..

[178] Zhang M., Michael L.H., Grosjean S.A., Kelly R.A., Carroll M.C., Entman M.L. (2006). The role of natural IgM in myocardial ischemia–reperfusion injury. J. Mol. Cell. Cardiol..

[179] Cain D.W., Cidlowski J.A. (2017). Immune regulation by glucocorticoids. Nat. Rev. Immunol..

[180] Libby P., Maroko P.R., Bloor C.M., Sobel B.E., Braunwald E. (1973). Reduction of experimental myocardial infarct size by corticosteroid administration. J. Clin. Investig..

[181] Roberts R., DeMello V., Sobel B.E. (1976). Deleterious effects of methylprednisolone in patients with myocardial infarction. Circulation.

[182] Kloner R.A., Fishbein M.C., Lew H., Maroko P.R., Braunwald E. (1978). Mummification of the infarcted myocardium by high dose corticosteroids. Circulation.

[183] Arriza J.L., Weinberger C., Cerelli G., Glaser T.M., Handelin B.L., Housman D.E., Evans R.M. (1987). Cloning of human mineralocorticoid receptor complementary DNA: Structural and functional kinship with the glucocorticoid receptor. Science.

[184] Usher M.G., Duan S.Z., Ivaschenko C.Y., Frieler R.A., Berger S., Schutz G., Lumeng C.N., Mortensen R.M. (2010). Myeloid mineralocorticoid receptor controls macrophage polarization and cardiovascular hypertrophy and remodeling in mice. J. Clin. Investig..

[185] Galuppo P., Vettorazzi S., Hovelmann J., Scholz C.J., Tuckermann J.P., Bauersachs J., Fraccarollo D. (2017). The glucocorticoid receptor in monocyte-derived macrophages is critical for cardiac infarct repair and remodeling. FASEB J..

[186] Metz C.A., Stubbs D.F., Hearron M.S. (1986). Significance of infarct site and methylprednisolone on survival following acute myocardial infarction. J. Int. Med. Res..

[187] Lefer A.M., Polansky E.W. (1979). Beneficial effects of ibuprofen in acute myocardial ischemia. Cardiology.

[188] Abbate A., Limana F., Capogrossi M.C., Santini D., Biondi-Zoccai G.G., Scarpa S., Germani A., Straino S., Severino A., Vasaturo F. (2006). Cyclo-oxygenase-2 (COX-2) inhibition reduces apoptosis in acute myocardial infarction. Apoptosis Int. J. Program. Cell Death.

[189] Brown E.J., Kloner R.A., Schoen F.J., Hammerman H., Hale S., Braunwald E. (1983). Scar thinning due to ibuprofen administration after experimental myocardial infarction. Am. J. Cardiol..

[190] Hammerman H., Alker K.J., Schoen F.J., Kloner R.A. (1984). Morphologic and functional effects of piroxicam on myocardial scar formation after coronary occlusion in dogs. Am. J. Cardiol..

[191] Timmers L., Sluijter J.P., Verlaan C.W., Steendijk P., Cramer M.J., Emons M., Strijder C., Grundeman P.F., Sze S.K., Hua L. (2007). Cyclooxygenase-2 inhibition increases mortality, enhances left ventricular remodeling, and impairs systolic function after myocardial infarction in the pig. Circulation.

[192] Gislason G.H., Jacobsen S., Rasmussen J.N., Rasmussen S., Buch P., Friberg J., Schramm T.K., Abildstrom S.Z., Kober L., Madsen M. (2006). Risk of death or reinfarction associated with the use of selective cyclooxygenase-2 inhibitors and nonselective nonsteroidal antiinflammatory drugs after acute myocardial infarction. Circulation.

[193] Brophy J.M., Levesque L.E., Zhang B. (2007). The coronary risk of cyclo-oxygenase-2 inhibitors in patients with a previous myocardial infarction. Heart.

[194] Schmidt M., Lamberts M., Olsen A.M., Fosboll E., Niessner A., Tamargo J., Rosano G., Agewall S., Kaski J.C., Kjeldsen K. (2016). Cardiovascular safety of non-aspirin non-steroidal anti-inflammatory drugs: Review and position paper by the working group for Cardiovascular Pharmacotherapy of the European Society of Cardiology. Eur. Heart J..

[195] Crompton M., Costi A. (1988). Kinetic evidence for a heart mitochondrial pore activated by Ca^2+^, inorganic phosphate and oxidative stress. A potential mechanism for mitochondrial dysfunction during cellular Ca^2+^ overload. Eur. J. Biochem..

[196] Lim W.Y., Messow C.M., Berry C. (2012). Cyclosporin variably and inconsistently reduces infarct size in experimental models of reperfused myocardial infarction: A systematic review and meta-analysis. Br. J. Pharmacol..

[197] Ong S.B., Samangouei P., Kalkhoran S.B., Hausenloy D.J. (2015). The mitochondrial permeability transition pore and its role in myocardial ischemia reperfusion injury. J. Mol. Cell Cardiol..

[198] Piot C., Croisille P., Staat P., Thibault H., Rioufol G., Mewton N., Elbelghiti R., Cung T.T., Bonnefoy E., Angoulvant D. (2008). Effect of cyclosporine on reperfusion injury in acute myocardial infarction. N. Engl. J. Med..

[199] Cung T.T., Morel O., Cayla G., Rioufol G., Garcia-Dorado D., Angoulvant D., Bonnefoy-Cudraz E., Guerin P., Elbaz M., Delarche N. (2015). Cyclosporine before PCI in Patients with Acute Myocardial Infarction. N. Engl. J. Med..

[200] Murry C.E., Jennings R.B., Reimer K.A. (1986). Preconditioning with ischemia: A delay of lethal cell injury in ischemic myocardium. Circulation.

[201] Heusch G., Botker H.E., Przyklenk K., Redington A., Yellon D. (2015). Remote ischemic conditioning. J. Am. Coll. Cardiol..

[202] Rossello X., Yellon D.M. (2018). The RISK pathway and beyond. Basic. Res. Cardiol..

[203] Wang Q., Liu G.P., Xue F.S., Wang S.Y., Cui X.L., Li R.P., Yang G.Z., Sun C., Liao X. (2015). Combined Vagal Stimulation and Limb Remote Ischemic Perconditioning Enhances Cardioprotection via an Anti-inflammatory Pathway. Inflammation.

[204] El Desoky E.S., Hassan A.K.M., Salem S.Y., Fadil S.A., Taha A.F. (2016). Cardioprotective effect of atorvastatin alone or in combination with remote ischemic preconditioning on the biochemical changes induced by ischemic/reperfusion injury in a mutual prospective study with a clinical and experimental animal arm. Int. J. Cardiol..

[205] Zhang J., Zhang J., Yu P., Chen M., Peng Q., Wang Z., Dong N. (2017). Remote Ischaemic Preconditioning and Sevoflurane Postconditioning Synergistically Protect Rats from Myocardial Injury Induced by Ischemia and Reperfusion Partly via Inhibition TLR4/MyD88/NF-kappaB Signaling Pathway. Cell Physiol. Biochem..

[206] Chen H., Jing X.Y., Shen Y.J., Wang T.L., Ou C., Lu S.F., Cai Y., Li Q., Chen X., Ding Y.J. (2018). Stat5-dependent cardioprotection in late remote ischaemia preconditioning. Cardiovasc. Res..

[207] Pilz P.M., Hamza O., Gidlof O., Goncalves I.F., Tretter E.V., Trojanek S., Abraham D., Heber S., Haller P.M., Podesser B.K. (2019). Remote ischemic perconditioning attenuates adverse cardiac remodeling and preserves left ventricular function in a rat model of reperfused myocardial infarction. Int. J. Cardiol..

[208] Gaspar A., Lourenco A.P., Pereira M.A., Azevedo P., Roncon-Albuquerque R., Marques J., Leite-Moreira A.F. (2018). Randomized controlled trial of remote ischaemic conditioning in ST-elevation myocardial infarction as adjuvant to primary angioplasty (RIC-STEMI). Basic Res. Cardiol..

[209] Hausenloy D.J., Kharbanda R.K., Moller U.K., Ramlall M., Aaroe J., Butler R., Bulluck H., Clayton T., Dana A., Dodd M. (2019). Effect of remote ischaemic conditioning on clinical outcomes in patients with acute myocardial infarction (CONDI-2/ERIC-PPCI): A single-blind randomised controlled trial. Lancet.

[210] Gong R., Wu Y.Q. (2019). Remote ischemic conditioning during primary percutaneous coronary intervention in patients with ST-segment elevation myocardial infarction: A systematic review and meta-analysis. J. Cardiothorac. Surg..

[211] Lindsey M.L., Brunt K.R., Kirk J.A., Kleinbongard P., Calvert J.W., de Castro Bras L.E., DeLeon-Pennell K.Y., Del Re D.P., Frangogiannis N.G., Frantz S. (2021). Guidelines for in vivo mouse models of myocardial infarction. Am. J. Physiol.-Heart Circ. Physiol..

[212] Gedik N., Kottenberg E., Thielmann M., Frey U.H., Jakob H., Peters J., Heusch G., Kleinbongard P. (2017). Potential humoral mediators of remote ischemic preconditioning in patients undergoing surgical coronary revascularization. Sci. Rep..

[213] Nederlof R., Weber N.C., Juffermans N.P., de Mol B.A., Hollmann M.W., Preckel B., Zuurbier C.J. (2017). A randomized trial of remote ischemic preconditioning and control treatment for cardioprotection in sevoflurane-anesthetized CABG patients. BMC Anesthesiol..

[214] Ney J., Hoffmann K., Meybohm P., Goetzenich A., Kraemer S., Benstom C., Weber N.C., Bickenbach J., Rossaint R., Marx G. (2018). Remote Ischemic Preconditioning Does Not Affect the Release of Humoral Factors in Propofol-Anesthetized Cardiac Surgery Patients: A Secondary Analysis of the RIPHeart Study. Int. J. Mol. Sci..

[215] Wang H., Lyu Y., Liao Q., Jin L., Xu L., Hu Y., Yu Y., Guo K. (2019). Effects of Remote Ischemic Preconditioning in Patients Undergoing Off-Pump Coronary Artery Bypass Graft Surgery. Front. Physiol..

[216] Yasojima K., Schwab C., McGeer E.G., McGeer P.L. (1998). Human heart generates complement proteins that are upregulated and activated after myocardial infarction. Circ. Res..

[217] Pischke S.E., Gustavsen A., Orrem H.L., Egge K.H., Courivaud F., Fontenelle H., Despont A., Bongoni A.K., Rieben R., Tonnessen T.I. (2017). Complement factor 5 blockade reduces porcine myocardial infarction size and improves immediate cardiac function. Basic. Res. Cardiol..

[218] Mahaffey K.W., Granger C.B., Nicolau J.C., Ruzyllo W., Weaver W.D., Theroux P., Hochman J.S., Filloon T.G., Mojcik C.F., Todaro T.G. (2003). Effect of pexelizumab, an anti-C5 complement antibody, as adjunctive therapy to fibrinolysis in acute myocardial infarction: The COMPlement inhibition in myocardial infarction treated with thromboLYtics (COMPLY) trial. Circulation.

[219] Granger C.B., Mahaffey K.W., Weaver W.D., Theroux P., Hochman J.S., Filloon T.G., Rollins S., Todaro T.G., Nicolau J.C., Ruzyllo W. (2003). Pexelizumab, an anti-C5 complement antibody, as adjunctive therapy to primary percutaneous coronary intervention in acute myocardial infarction: The COMplement inhibition in Myocardial infarction treated with Angioplasty (COMMA) trial. Circulation.

[220] Investigators A.A., Armstrong P.W., Granger C.B., Adams P.X., Hamm C., Holmes D., O’Neill W.W., Todaro T.G., Vahanian A., Van de Werf F. (2007). Pexelizumab for acute ST-elevation myocardial infarction in patients undergoing primary percutaneous coronary intervention: A randomized controlled trial. JAMA.

[221] Hayashidani S., Tsutsui H., Shiomi T., Ikeuchi M., Matsusaka H., Suematsu N., Wen J., Egashira K., Takeshita A. (2003). Anti-monocyte chemoattractant protein-1 gene therapy attenuates left ventricular remodeling and failure after experimental myocardial infarction. Circulation.

[222] Montecucco F., Braunersreuther V., Lenglet S., Delattre B.M., Pelli G., Buatois V., Guilhot F., Galan K., Vuilleumier N., Ferlin W. (2012). CC chemokine CCL5 plays a central role impacting infarct size and post-infarction heart failure in mice. Eur. Heart J..

[223] Leuschner F., Dutta P., Gorbatov R., Novobrantseva T.I., Donahoe J.S., Courties G., Lee K.M., Kim J.I., Markmann J.F., Marinelli B. (2011). Therapeutic siRNA silencing in inflammatory monocytes in mice. Nat. Biotechnol..

[224] Dobaczewski M., Xia Y., Bujak M., Gonzalez-Quesada C., Frangogiannis N.G. (2010). CCR5 signaling suppresses inflammation and reduces adverse remodeling of the infarcted heart, mediating recruitment of regulatory T cells. Am. J. Pathol..

[225] Simpson P.J., Todd R.F., Fantone J.C., Mickelson J.K., Griffin J.D., Lucchesi B.R. (1988). Reduction of experimental canine myocardial reperfusion injury by a monoclonal antibody (anti-Mo1, anti-CD11b) that inhibits leukocyte adhesion. J. Clin. Investig..

[226] Ma X.L., Tsao P.S., Lefer A.M. (1991). Antibody to CD-18 exerts endothelial and cardiac protective effects in myocardial ischemia and reperfusion. J. Clin. Investig..

[227] Aversano T., Zhou W., Nedelman M., Nakada M., Weisman H. (1995). A chimeric IgG4 monoclonal antibody directed against CD18 reduces infarct size in a primate model of myocardial ischemia and reperfusion. J. Am. Coll. Cardiol..

[228] Sager H.B., Dutta P., Dahlman J.E., Hulsmans M., Courties G., Sun Y., Heidt T., Vinegoni C., Borodovsky A., Fitzgerald K. (2016). RNAi targeting multiple cell adhesion molecules reduces immune cell recruitment and vascular inflammation after myocardial infarction. Sci. Transl. Med..

[229] Baran K.W., Nguyen M., McKendall G.R., Lambrew C.T., Dykstra G., Palmeri S.T., Gibbons R.J., Borzak S., Sobel B.E., Gourlay S.G. (2001). Limitation of Myocardial Infarction Following Thrombolysis in Acute Myocardial Infarction Study, Double-blind, randomized trial of an anti-CD18 antibody in conjunction with recombinant tissue plasminogen activator for acute myocardial infarction: Limitation of myocardial infarction following thrombolysis in acute myocardial infarction (LIMIT AMI) study. Circulation.

[230] Faxon D.P., Gibbons R.J., Chronos N.A., Gurbel P.A., Sheehan F., Investigators H.-M. (2002). The effect of blockade of the CD11/CD18 integrin receptor on infarct size in patients with acute myocardial infarction treated with direct angioplasty: The results of the HALT-MI study. J. Am. Coll. Cardiol..

[231] Rusnak J.M., Kopecky S.L., Clements I.P., Gibbons R.J., Holland A.E., Peterman H.S., Martin J.S., Saoud J.B., Feldman R.L., Breisblatt W.M. (2001). An anti-CD11/CD18 monoclonal antibody in patients with acute myocardial infarction having percutaneous transluminal coronary angioplasty (the FESTIVAL study). Am. J. Cardiol..

[232] Tardif J.C., Tanguay J.F., Wright S.R., Duchatelle V., Petroni T., Gregoire J.C., Ibrahim R., Heinonen T.M., Robb S., Bertrand O.F. (2013). Effects of the P-selectin antagonist inclacumab on myocardial damage after percutaneous coronary intervention for non-ST-segment elevation myocardial infarction: Results of the SELECT-ACS trial. J. Am. Coll. Cardiol..

[233] Abbate A., Salloum F.N., Vecile E., Das A., Hoke N.N., Straino S., Biondi-Zoccai G.G., Houser J.E., Qureshi I.Z., Ownby E.D. (2008). Anakinra, a recombinant human interleukin-1 receptor antagonist, inhibits apoptosis in experimental acute myocardial infarction. Circulation.

[234] Toldo S., Mezzaroma E., Van Tassell B.W., Farkas D., Marchetti C., Voelkel N.F., Abbate A. (2013). Interleukin-1beta blockade improves cardiac remodelling after myocardial infarction without interrupting the inflammasome in the mouse. Exp. Physiol..

[235] Abbate A., Trankle C.R., Buckley L.F., Lipinski M.J., Appleton D., Kadariya D., Canada J.M., Carbone S., Roberts C.S., Abouzaki N. (2020). Interleukin-1 Blockade Inhibits the Acute Inflammatory Response in Patients with ST-Segment-Elevation Myocardial Infarction. J. Am. Heart Assoc..

[236] Abbate A., Van Tassell B.W., Biondi-Zoccai G., Kontos M.C., Grizzard J.D., Spillman D.W., Oddi C., Roberts C.S., Melchior R.D., Mueller G.H. (2013). Effects of interleukin-1 blockade with anakinra on adverse cardiac remodeling and heart failure after acute myocardial infarction [from the Virginia Commonwealth University-Anakinra Remodeling Trial (2) (VCU-ART2) pilot study]. Am. J. Cardiol..

[237] Abbate A., Kontos M.C., Grizzard J.D., Biondi-Zoccai G.G., Van Tassell B.W., Robati R., Roach L.M., Arena R.A., Roberts C.S., Varma A. (2010). Interleukin-1 blockade with anakinra to prevent adverse cardiac remodeling after acute myocardial infarction (Virginia Commonwealth University Anakinra Remodeling Trial [VCU-ART] Pilot study). Am. J. Cardiol..

[238] Abbate A., Kontos M.C., Abouzaki N.A., Melchior R.D., Thomas C., Van Tassell B.W., Oddi C., Carbone S., Trankle C.R., Roberts C.S. (2015). Comparative safety of interleukin-1 blockade with anakinra in patients with ST-segment elevation acute myocardial infarction (from the VCU-ART and VCU-ART2 pilot studies). Am. J. Cardiol..

[239] Ridker P.M., Everett B.M., Thuren T., MacFadyen J.G., Chang W.H., Ballantyne C., Fonseca F., Nicolau J., Koenig W., Anker S.D. (2017). Antiinflammatory Therapy with Canakinumab for Atherosclerotic Disease. N. Engl. J. Med..

[240] Ridker P.M., MacFadyen J.G., Everett B.M., Libby P., Thuren T., Glynn R.J., Group C.T. (2018). Relationship of C-reactive protein reduction to cardiovascular event reduction following treatment with canakinumab: A secondary analysis from the CANTOS randomised controlled trial. Lancet.

[241] Everett B.M., Cornel J.H., Lainscak M., Anker S.D., Abbate A., Thuren T., Libby P., Glynn R.J., Ridker P.M. (2019). Anti-Inflammatory Therapy with Canakinumab for the Prevention of Hospitalization for Heart Failure. Circulation.

[242] Broz P., Dixit V.M. (2016). Inflammasomes: Mechanism of assembly; regulation; signalling. Nat. Rev. Immunol..

[243] Toldo S., Mauro A.G., Cutter Z., Van Tassell B.W., Mezzaroma E., Del Buono M.G., Prestamburgo A., Potere N., Abbate A. (2019). The NLRP_3_ Inflammasome Inhibitor, OLT_1177_ (Dapansutrile), Reduces Infarct Size and Preserves Contractile Function After Ischemia Reperfusion Injury in the Mouse. J. Cardiovasc. Pharmacol..

[244] Wohlford G.F., Van Tassell B.W., Billingsley H.E., Kadariya D., Canada J.M., Carbone S., Mihalick V.L., Bonaventura A., Vecchie A., Chiabrando J.G. (2020). Phase 1B, Randomized, Double-Blinded, Dose Escalation, Single-Center, Repeat Dose Safety and Pharmacodynamics Study of the Oral NLRP3 Inhibitor Dapansutrile in Subjects With NYHA II-III Systolic Heart Failure. J. Cardiovasc. Pharmacol..

[245] Tardif J.C., Kouz S., Waters D.D., Bertrand O.F., Diaz R., Maggioni A.P., Pinto F.J., Ibrahim R., Gamra H., Kiwan G.S. (2019). Efficacy and Safety of Low-Dose Colchicine after Myocardial Infarction. N. Engl. J. Med..

[246] Cronstein B.N., Molad Y., Reibman J., Balakhane E., Levin R.I., Weissmann G. (1995). Colchicine alters the quantitative and qualitative display of selectins on endothelial cells and neutrophils. J. Clin. Investig..

[247] Martinon F., Petrilli V., Mayor A., Tardivel A., Tschopp J. (2006). Gout-associated uric acid crystals activate the NALP3 inflammasome. Nature.

[248] Deftereos S., Giannopoulos G., Angelidis C., Alexopoulos N., Filippatos G., Papoutsidakis N., Sianos G., Goudevenos J., Alexopoulos D., Pyrgakis V. (2015). Anti-Inflammatory Treatment with Colchicine in Acute Myocardial Infarction: A Pilot Study. Circulation.

[249] Markousis-Mavrogenis G., Tromp J., Ouwerkerk W., Devalaraja M., Anker S.D., Cleland J.G., Dickstein K., Filippatos G.S., van der Harst P., Lang C.C. (2019). The clinical significance of interleukin-6 in heart failure: Results from the BIOSTAT-CHF study. Eur. J. Heart Fail..

[250] George M.J., Jasmin N.H., Cummings V.T., Richard-Loendt A., Launchbury F., Woollard K., Turner-Stokes T., Diaz A.I.G., Lythgoe M., Stuckey D.J. (2021). Selective Interleukin-6 Trans-Signaling Blockade Is More Effective Than Panantagonism in Reperfused Myocardial Infarction. JACC Basic. Transl. Sci..

[251] Kleveland O., Kunszt G., Bratlie M., Ueland T., Broch K., Holte E., Michelsen A.E., Bendz B., Amundsen B.H., Espevik T. (2016). Effect of a single dose of the interleukin-6 receptor antagonist tocilizumab on inflammation and troponin T release in patients with non-ST-elevation myocardial infarction: A double-blind, randomized, placebo-controlled phase 2 trial. Eur. Heart J..

[252] Broch K., Anstensrud A.K., Woxholt S., Sharma K., Tollefsen I.M., Bendz B., Aakhus S., Ueland T., Amundsen B.H., Damas J.K. (2021). Randomized Trial of Interleukin-6 Receptor Inhibition in Patients With Acute ST-Segment Elevation Myocardial Infarction. J. Am. Coll. Cardiol..

[253] Huse C., Anstensrud A.K., Michelsen A.E., Ueland T., Broch K., Woxholt S., Yang K., Sharma K., Tollefsen I.M., Bendz B. (2022). Interleukin-6 inhibition in ST-elevation myocardial infarction: Immune cell profile in the randomised ASSAIL-MI trial. EBioMedicine.

[254] Frangogiannis N.G. (2014). The inflammatory response in myocardial injury, repair, and remodelling, Nature reviews. Cardiology.

[255] Shiraishi M., Shintani Y., Shintani Y., Ishida H., Saba R., Yamaguchi A., Adachi H., Yashiro K., Suzuki K. (2016). Alternatively activated macrophages determine repair of the infarcted adult murine heart. J. Clin. Investig..

[256] Garcia R.A., Lupisella J.A., Ito B.R., Hsu M.Y., Fernando G., Carson N.L., Allocco J.J., Ryan C.S., Zhang R., Wang Z. (2021). Selective FPR2 Agonism Promotes a Proresolution Macrophage Phenotype and Improves Cardiac Structure-Function Post Myocardial Infarction. JACC Basic. Transl. Sci..

